# Clusterization in acute myeloid leukemia based on prognostic alternative splicing signature to reveal the clinical characteristics in the bone marrow microenvironment

**DOI:** 10.1186/s13578-020-00481-5

**Published:** 2020-10-12

**Authors:** Nan Zhang, Ping Zhang, Ying Chen, Shifeng Lou, Hanqing Zeng, Jianchuan Deng

**Affiliations:** 1 Department of Hematology, The Second Affiliated Hospital of Chongqing Medical University, 76 Linjiang Road, Chongqing, 400010 People’s Republic of China; 2 Hematology Laboratory, The Second Affiliated Hospital of Chongqing Medical University, Chongqing, 400010 China

**Keywords:** Acute myeloid leukemia, Alternative splicing, Prognosis, Microenvironment, Genome-wide analysis

## Abstract

**Background:**

Alternative splicing (AS), a crucial post-transcriptional regulatory mechanism in expanding the coding capacities of genomes and increasing the diversity of proteins, still faces various challenges in the splicing regulation mechanism of acute myeloid leukemia (AML) and microenvironmental changes.

**Results:**

A total of 27,833 AS events were detected in 8337 genes in 178 AML patients, with exon skip being the predominant type. Approximately 11% of the AS events were significantly related to prognosis, and the prediction models based on various events demonstrated high classification efficiencies. Splicing factors correlation networks further altered the diversity of AS events through epigenetic regulation and clarified the potential mechanism of the splicing pathway. Unsupervised cluster analysis revealed significant correlations between AS and immune features, molecular mutations, immune checkpoints and clinical outcome. The results suggested that AS clusters could be used to identify patient subgroups with different survival outcomes in AML, among which C1 was both associated with good outcome in overall survival. Interestingly, C1 was associated with lower immune scores compared with C2 and C3, and favorable-risk cytogenetics was rarely distributed in C2, but much more common in C1.

**Conclusions:**

This study revealed a comprehensive landscape of AS events, and provides new insight into molecular targeted therapy and immunotherapy strategy for AML.

## Background

The clinical of acute myeloid leukemia (AML) from initial diagnosis to recurrent/refractory status is characterized by the acquisition of drug resistance and progressive immune dysfunction [1]. Chemotherapy, target drugs, hematopoietic stem cell transplantation, and immunotherapy are the current treatment options for patients with AML [2]. The 5-year relative survival rate in adolescents is 66%, but it declines to 54%, 32%, and 7% among patients aged 20–49 years, 50–64 years, and over 65 years, respectively [3]. Despite significant progresses in diagnostic approach and therapeutic strategies in the recent years, few patients could benefit from these advances [4]. Recent studies indicated that several phenotypic and genetic changes in bone marrow microenvironment could support the genesis of leukemia, facilitate leukemic cells survival and mediate chemotherapy resistance [5, 6]. Therefore, the molecular mechanisms of AML and the related microenvironmental regulation are still critical issues.

With recent developments in next generation sequencing techniques, molecular evolutionary mechanisms underlying the occurrence and clinical progression of AML are better understood, and could optimize individualized treatment [7, 8]. Alternative splicing (AS) is a crucial post-transcriptional regulatory mechanism that converts only 20,000 genes in eukaryotic cells into approximately 95,000 different proteins for generating protein diversity [9]. Recent studies have found that variations in tumor transcriptome due to AS changes and dysregulation of AS were closely related to tumor progression, drug resistance, metastasis and other carcinogenic processes [10]. Additionally, occurrence of some peculiar AS events may represent the phenotypic characteristics of specific malignancies. Ghigna et al. identified that the exon skip events of *MST1R* was related to acquisition of cellular motility during cell invasion [11]. Bechara et al. suggested that changes in an exon of *NUMB* can activate proliferation of lung cancer cells [12]. Nevertheless, the molecular mechanism of AS mainly depends on the regulation of splicing factors [13]. Tumor genome sequencing data demonstrated that multiple mutations have been found in spliceosomal complex members, while common in hematologic malignancies and usually occurs in *SRSF2*, *SF3B1*, *ZRSR2* and *U2AF1* [14]. Therefore, a good understanding of the potential function of AS could help researchers to identify new oncogenic mechanisms and therapeutic targets.

In recent decades, significant advances have been achieved in the field of tumor immunotherapy [15, 16]. Bone marrow microenvironment has been identified as a major sanctuary of leukemic stem cells (LSCs) to protect these cells from conventional therapies, immune cells, hematopoietic cells and some cytokines, chemokines, etc. [17]. Moreover, increasing evidence suggested that AS is another hallmark in immune microenvironment formation [18]. Studies have shown that splicing changes can be used to distinguish different types or subtypes of tumors and are related to clinical stages and prognosis [19, 20]. Therefore, better evaluation of bone marrow microenvironment and distribution and function of splicing events are essential to improve the efficacy of immunotherapy.

Currently, RNA-seq for detecting AS events is frequently used, and specific implementation focuses on bioinformatics analysis of data [21]. With rapid accumulation of sequencing data, public databases provide wealthy resources for systemic biology. In this study, we systematically assessed genome-wide AS patterns and evaluated their associations with clinical outcomes of AML. Furthermore, we discerned distinct clusters of AML based on survival-associated events and investigated the relationship between clusters and clinical characteristics of bone marrow immune microenvironment. Our study revealed a comprehensive landscape of AS events, and provided new insight into molecular targeted therapy and immunotherapy strategy for AML.

## Results

### Overview of AS events in AML

The profile of AS events for 178 patients with AML were analyzed from TCGA cohort. The clinical and molecular characteristics of these cases were summarized in Additional file 1: Table S1. The median age at diagnosis was 55 years (range, 18 to 88), and the median follow-up duration was 16.4 months (range, 1 to 118).

**Fig. 1:**
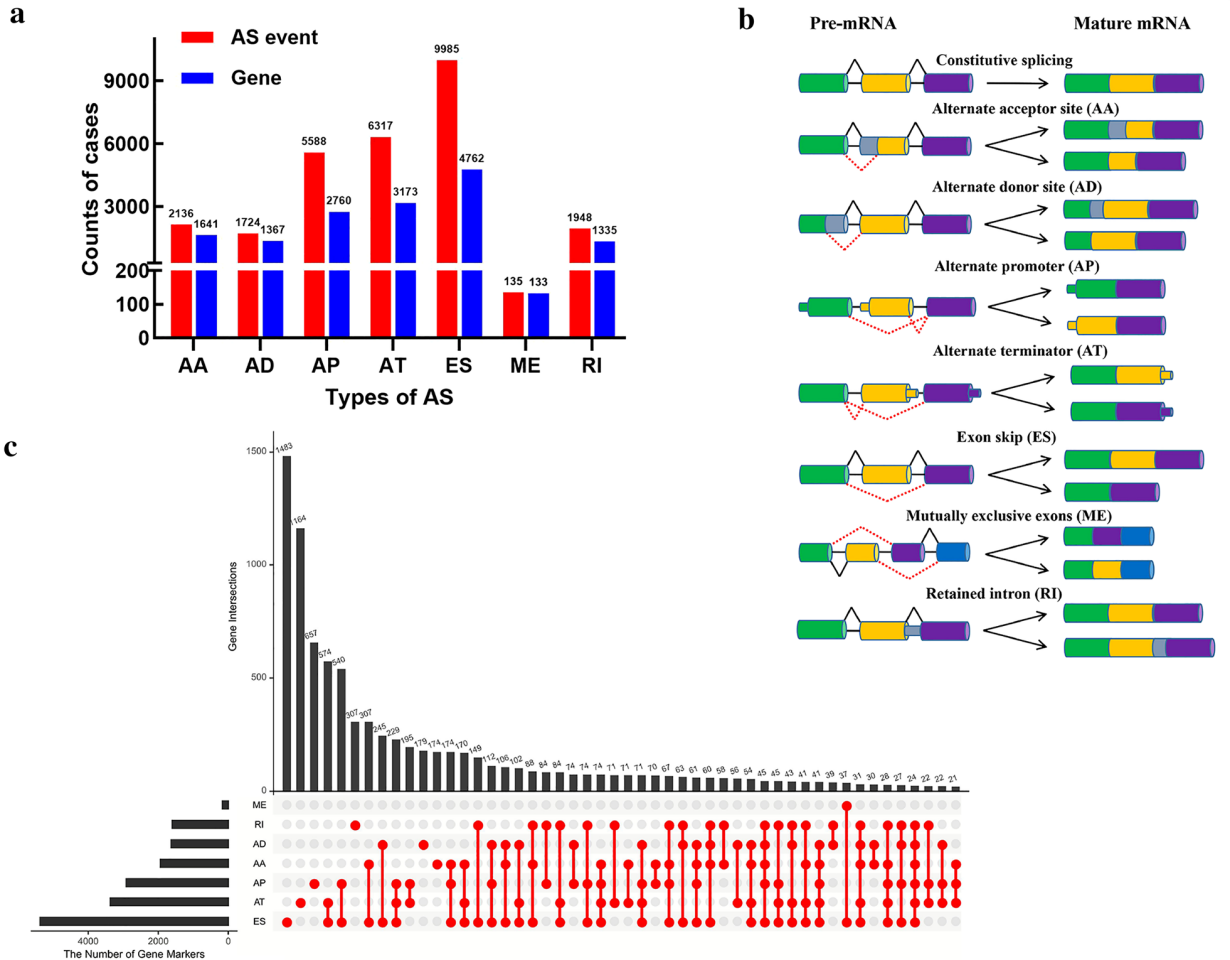
Overview of AS events profiling in AML. **a** Number of AS events and parent genes from 178 patients with AML. **b** Schematic representation for seven types of AS events. *AA* Alternate Acceptor site, *AD* Alternate Donor site, *AP* Alternate Promoter, *AT* Alternate Terminator, *ES* Exon Skip, *ME* Mutually Exclusive Exons, *RI* Retained Intron. **c** Upset plot of parent gene interactions between the seven types of AS events in AML

After preprocessing procedure, integrated RNA-Seq data with AS events in seven splicing types were included in the present study. We detected a total of 27,833 AS events of 8,337 genes, comprising 2,136 AA in 1641 genes, 1724 AD in 1367 genes, 5588 AP in 2760 genes, 6317 AT in 3173 genes, 9985 ES in 4762 genes, 135 ME in 133 genes, 1948 RI in 1335 genes, as illustrated in Fig. 1a. A schematic diagram of AS events are shown in Fig. 1b. It should be noted that several mRNA splicing events may be detected in a single gene, and up to 6 types of AS events were observable for one gene (Fig. 1c). In addition, most genes have more than one type of AS events. ES was the predominant type of AS events in AML (36.12%), and these events may provide perhaps a critical process for enriching transcriptome diversity.

### AS events associated with prognosis of AML

**Fig. 2:**
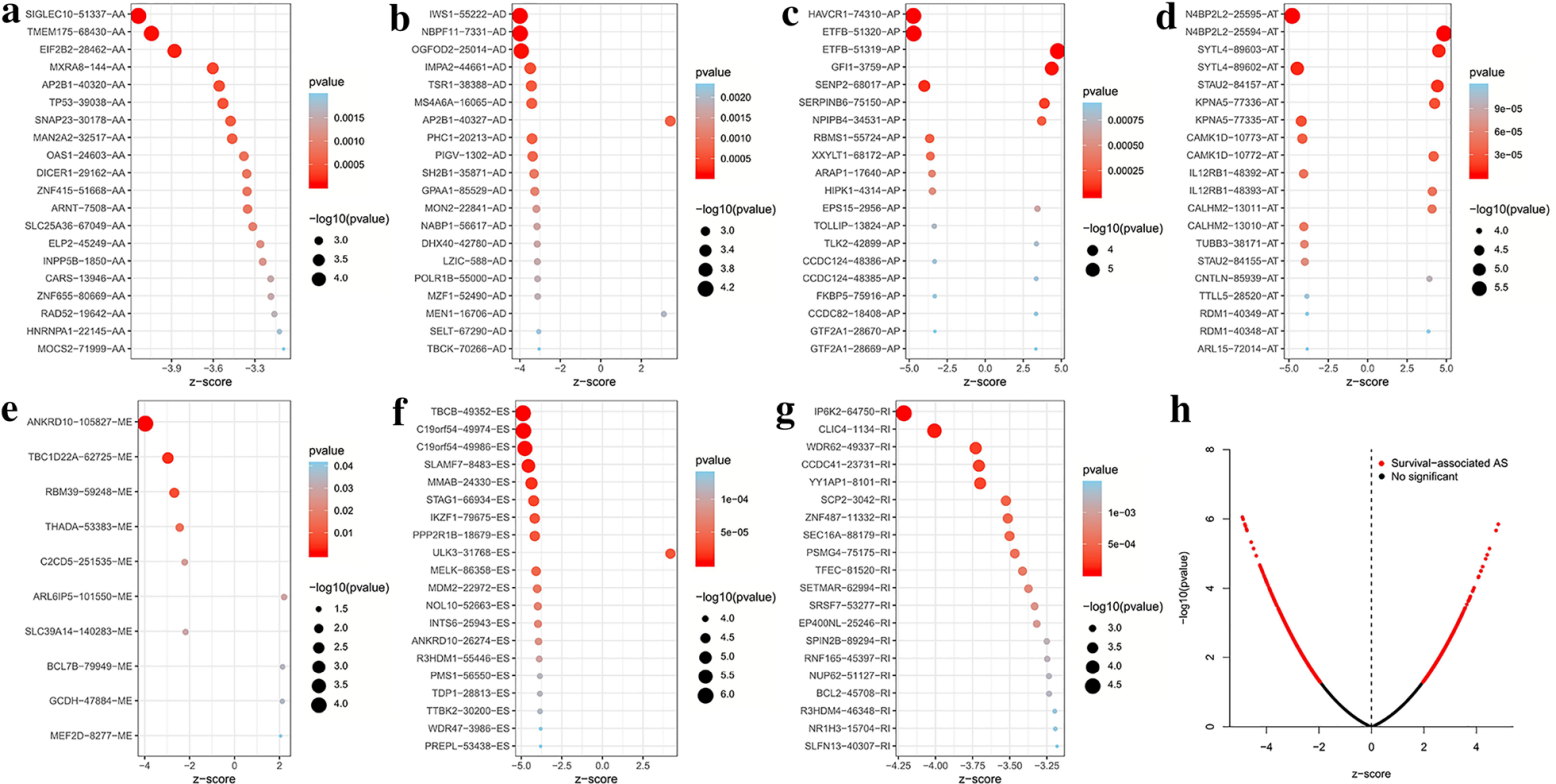
Survival-associated AS events in AML patients. **a-g** Bubble plots of the top 20 significant survival-associated AS events for AA, AD, AP, AT, ME, ES and RI. **h** Volcano plot of survival-associated AS events (red dot) and survival-irrelated AS events (black dot)

We performed univariate Cox analysis to evaluate the impact of AS type on OS in AML patients. A total of 3016 survival-related AS events within 1992 genes were identified. Hazard ratios (HRs) greater than or less than 1 accounted for 2.74% and 5.88% of the total AS events, respectively (Additional file 2: Table S2). Meanwhile, several survival-associated AS events can also be detected in one single gene, and different spliceosomes of a few parent genes (such as *NBPF11*, *LRRC23*, etc.) exhibited opposite prognostic effects in AML (Additional file 3: Figure S1). Top 20 most significant survival-associated AS events of each type were presented in Fig. 2.

Subsequently, independent prognostic AS events for seven types were used to construct prognostic predictors models. LASSO analysis was used to screen the optimal combination (Additional file 9: Figure S3). The Kaplan–Meier analysis indicated that these molecular signatures of AS events can be used to differentiate patients with distinct prognosis. To compare the efficiency of eight predictive models, ROC curves were applied into each model. The AUC of AA, AD, ES, AT, AP, RI, ME and marge-AS models was 0.912, 0.875, 0.834, 0.829, 0.820, 0.819, 0.738, and 0.867, respectively. AA was related with better predictive performance in AML, showed in Additional file 4: Figure S2. Detailed data of models based on each type of AS signature is listed in Additional file 5: Table S3.

### Clinical characteristics of bone marrow immune microenvironment

**Fig. 3:**
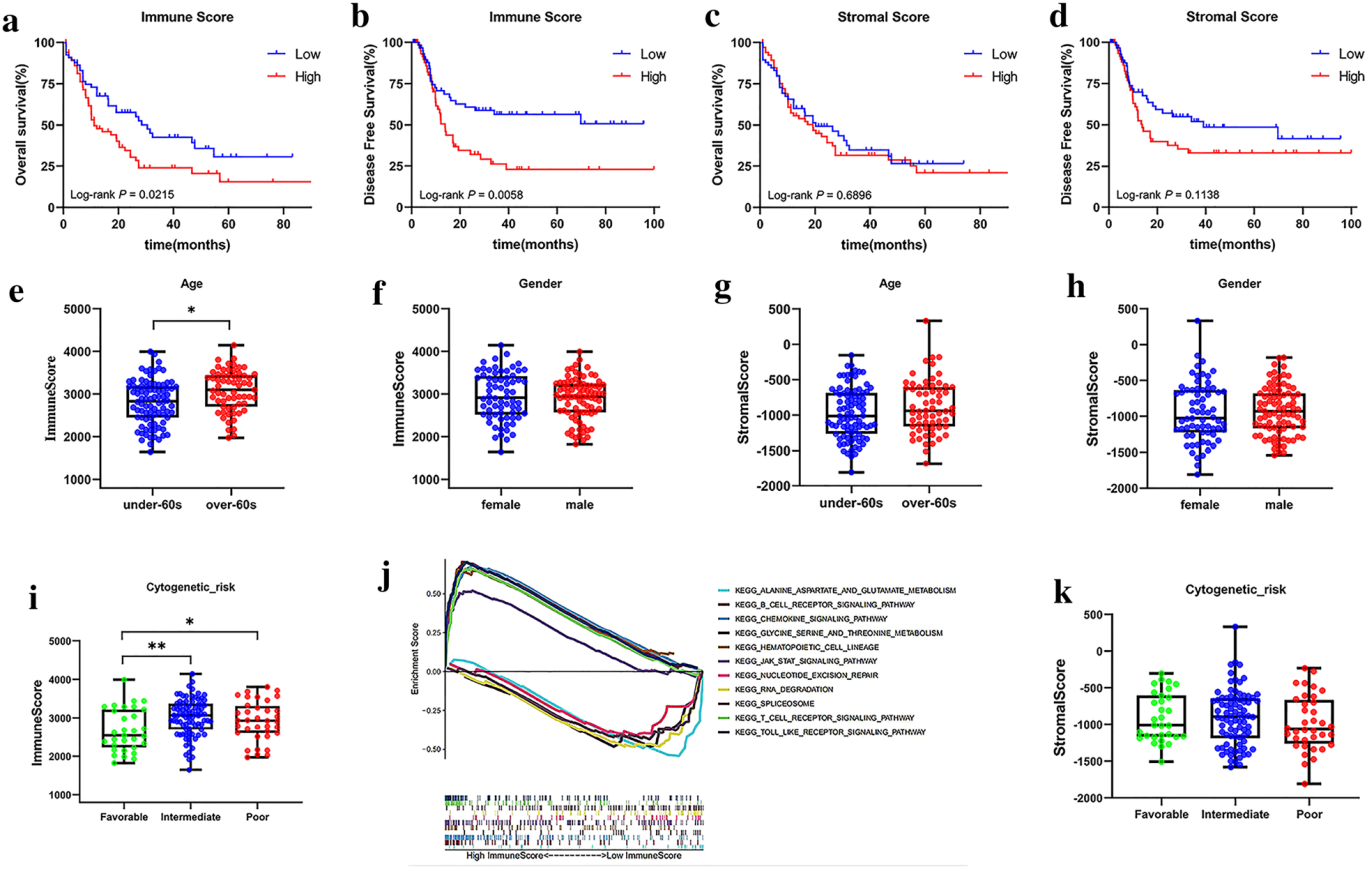
Immune scores and stromal scores are associated with clinical features in AML. **a-b** Kaplan–Meier curves of OS and DFS for patients with high vs. low immune scores. **c-d** Kaplan–Meier curves of OS and DFS for patients with high vs. low stromal scores. (**e–f**, **i**) Distribution of immune scores for age (under 60 yrs vs. over 60 yrs), gender (female vs. male) and cytogenetic risk classification (favorable, intermediate, and poor). (**g-h**, **k**) Distribution of stromal scores for age, gender and cytogenetic risk classification. **j** GSEA delineates biological pathways correlated with immune scores. Several enrichment results with significant associations between high- and low-immune scores groups are shown

To evaluate the relationship between AS and bone marrow microenvironment, we analyzed immune scores of AML patients by using a bioinformatics tool. Immune scores ranged from 1645.34 to 4145.87, and stromal scores form −1809.22 to 333.04. To assess the correlation of OS and DFS with scores, we divided patients into low- and high-score groups using the median of immune/stromal scores as cutoff. As shown in Fig. 3, the survival distribution curves showed that high immune scores correlated with worse OS (*P* value = 0.0215) and DFS (*P*-value = 0.0058) than low immune scores (Fig. 3a, b). However, patients with lower stromal scores only had numerically longer OS (*P*-value = 0.6896) and DFS (*P*-value = 0.1138) (Figure c, d). Since previous studies have indicated that immune conditions are associated with clinical characteristics, we investigated the relationships of gender, age and cytogenetic risk classification with immune and stromal scores. Immune scores were greater in the elderly group (*P*-value = 0.0013), while stromal scores were independent of age (*P*-value = 0.1196) and gender (P-value = 0.5156) (Fig. 3e-h). Intriguingly, the average immune scores of the favorable cytogenetics subtypes ranked the lowest in risk classification, which is consistent with findings above (Fig. 3i, k). To reveal the biological basis involved in different bone marrow microenvironments, we performed GSEA analysis in the high and low immune scores groups. Multiple pathways (Fig. 3J), including RNA degradation, nucleotide excision repair, hematopoietic cell lineage, and several metabolism related pathways were identified.

### Network of survival-associated AS events and splicing factors

**Fig. 4:**
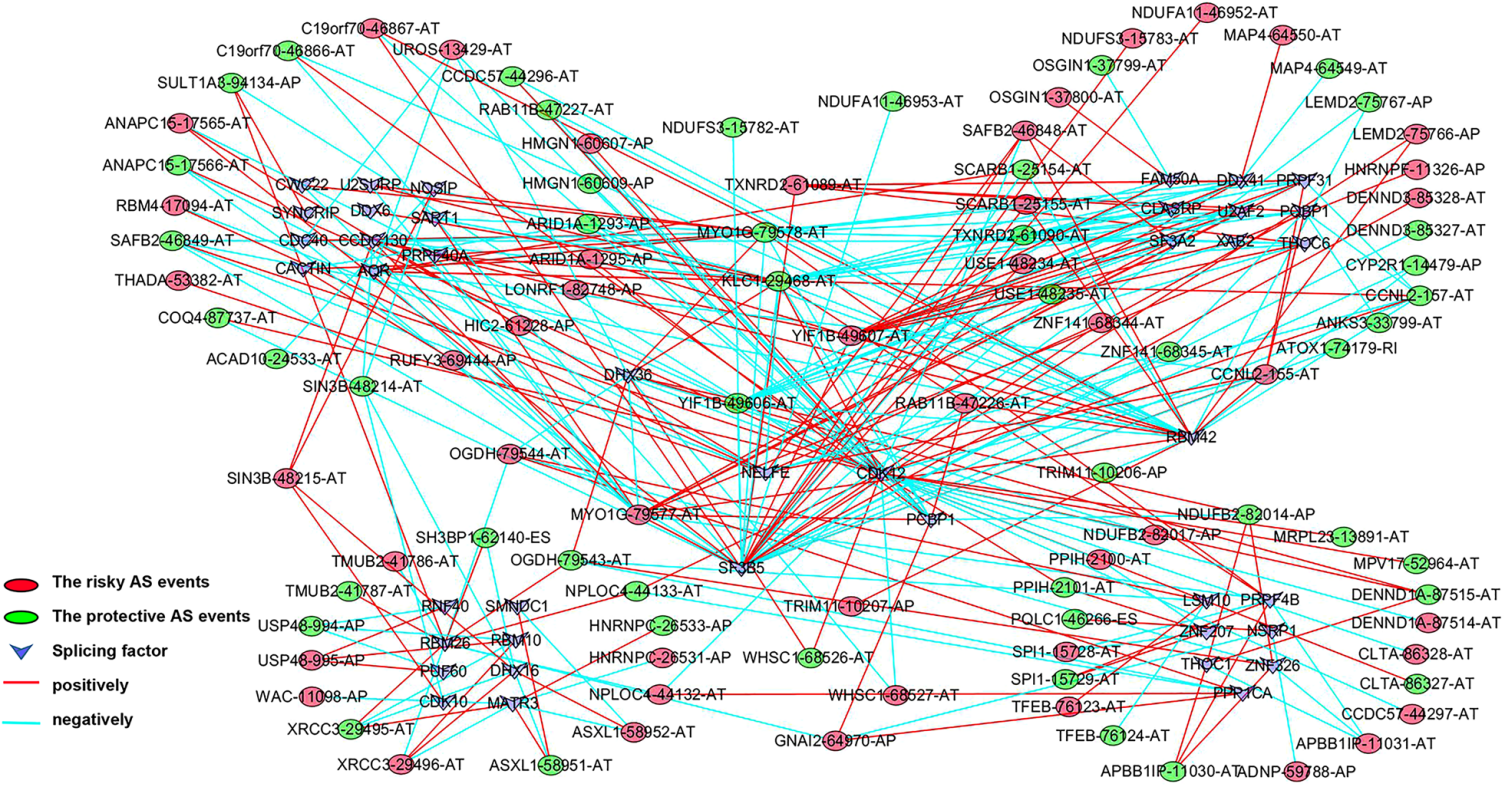
Correlation network of splicing factors (SFs) and the PSI values of survival-associated AS events in AML. A node represents a splicing factor or AS event, which is distinguished by the shape of the node, the color of the lines represents the trend of SFs regulation

**Fig. 5:**
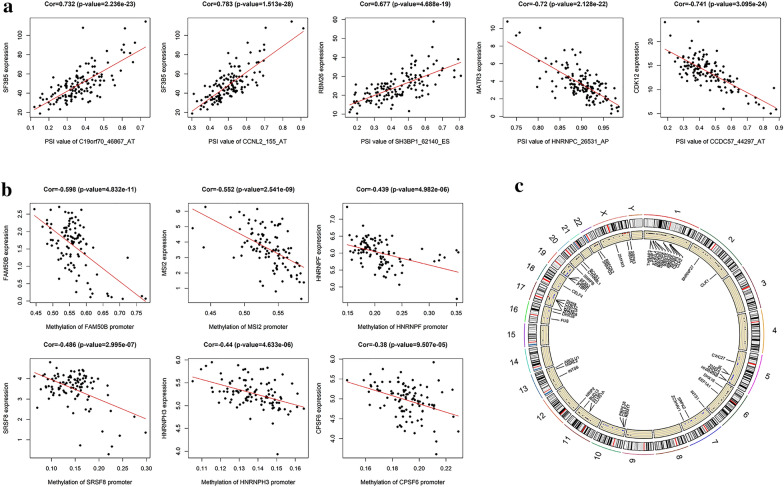
A representative diagram of the regulatory splicing events in AML. **a** Representative dot plots of the relationship between SFs expression and PSI values of AS events. **b** Representative dot plots of the relationship between SFs promoter methylation and SFs expression. **c** Representative dot plots of the chromosome position information of SFs

Splicing factors (SFs) are the key players of AS events and promote differential splicing patterns under stress conditions [22]. To understand the regulatory pattern of SFs in AML, we constructed the interaction networks of OS-related AS events and SFs (Fig. 4). A total of 117 AS events including 56 AS events with inferior prognosis (red dots) and 61 AS event with protective activity (green dots) were identified and correlated significantly with the levels of SF expression (purple triangle). Figure 5a shows several examples of the relationship between SF expression levels and PSI values for AS events, with more details provided in Additional file 6: Table S4. We observed that many SFs were correlated with different type of AS events and may have opposite effects, indicating that SFs may negatively (green line) or positively (red line) regulates OS-related AS events. Similarly, partial AS events could be regulated by multiple SFs simultaneously, this phenomenon partly explains the multiple AS events that can occur in the same transcript.

Since abnormal expression of SFs in cancer is a potential mechanism for regulating AS events, we analyzed the relationship between the observed changes in SFs expression and promoter methylation. Among the 89 SFs, the promoters of 72 SFs were highly hypermethylated and negatively correlated with their corresponding mRNA expression in AML patients. Figure 5b shows some highly relevant examples including *FAM50B*, *SRSF8*, *HNRNPF*, *CPSF6*, etc., with more details provided in Additional file 7: Table S5. In addition, we observed that 19.46% of cases in the AML cohort had at least one SF copy number variation. Circos plot shows the chromosome position information of partial SFs (Fig. 5c). This result revealed that SFs could be regulated by epigenetics, and further increase the diversity of AS events.

### AS-based clustering was associated with clinical characteristics and immune features

**Fig. 6:**
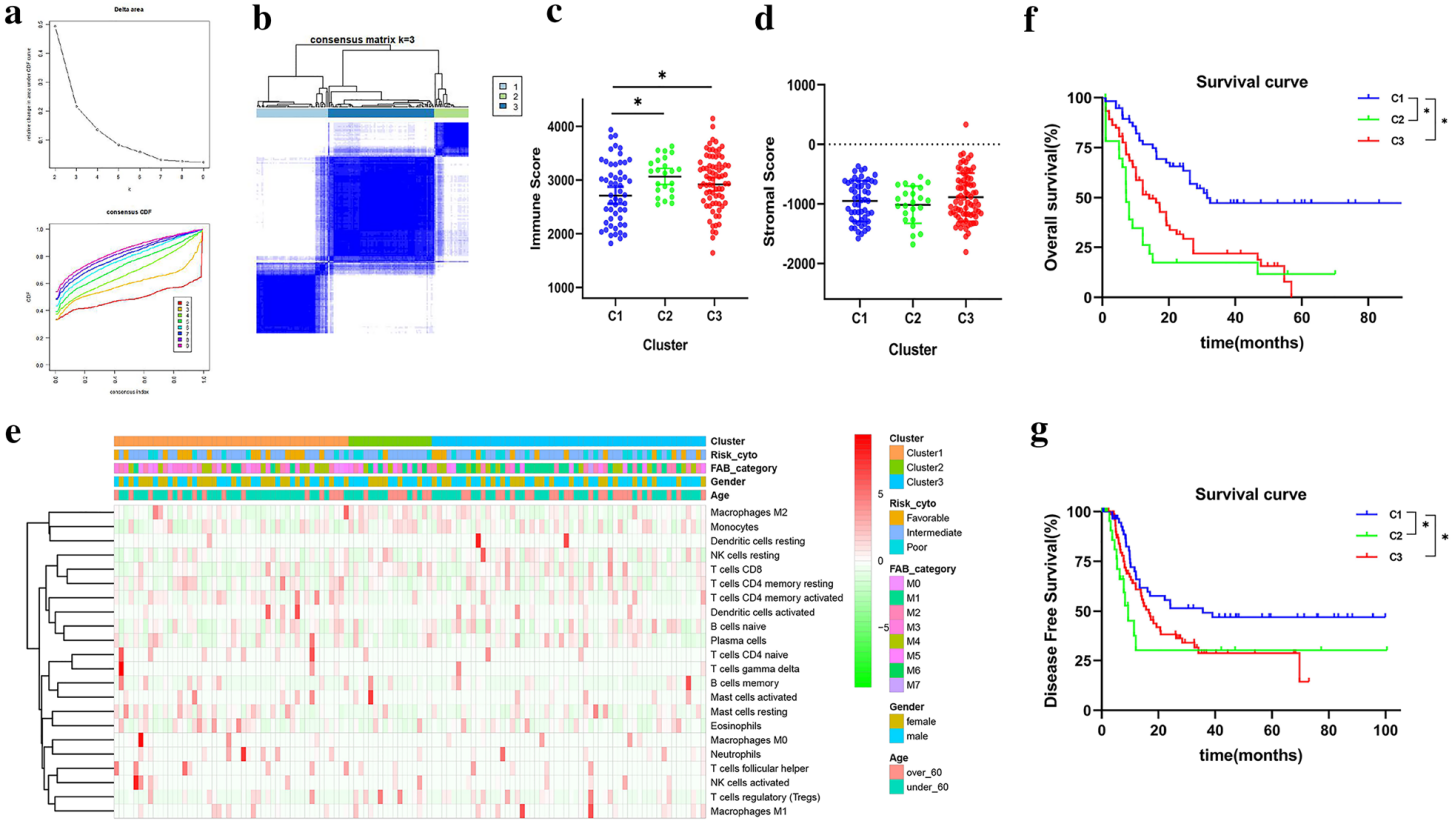
AS-based clustering is associated with clinical characteristics and immune features. **a** Elbow method and Gap statistic method analysis for different numbers of clusters (k = 2 to 9). **b** Consensus matrix heatmap defined three clusters of samples, C1 (n = 60, 33.7%), C2 (n = 29, 16.3%), C3 (n = 89, 50.0%). **c, d** Distribution of immune scores and stromal scores between three clusters. **e** Heatmap of clinical characteristics (gender, age, cytogenetics risk category, FAB subtype, immune features) between three clusters. **f, g** Kaplan–Meier curves of OS and DFS for three AS-based clusters. Depicted *P*-values are from log-rank tests

Our research observed that the PSI values of each AS event varied among AML individuals. To better understand the molecular heterogeneity of AML, we explored patterns of AS based on survival-associated events by performing consensus unsupervised analysis of all samples. As a result, three clusters of samples were determined (Fig. 6a, b): C1 (n = 60, 33.7%), C2 (n = 29, 16.3%), C3 (n = 89, 50.0%). To discern the characteristics of different subtypes, we first investigated the relationships between AS clusters and immune microenvironment. The results showed that C1 was associated with lower immune scores compared with C2 and C3 (C1 vs. C2, *P*-value = 0.0188; C1 vs. C3, *P*-value = 0.0463), see Fig. 6c. However, no significant difference was found between clusters and stromal scores (Fig. 6d). Additionally, we compared clinical characteristics (gender, age, cytogenetics risk category, FAB subtype) between clusters. As showed in Fig. 6e, favorable-risk cytogenetics was rarely distributed in C2 (3.57%), but much more common in C1 (30.0%). To explore the association between AS clusters and immune features, the landscape of 22 immune cells abundance within the bone marrow microenvironment of each AML case were plotted. Furthermore, Kaplan–Meier analysis was performed to assess the relationship between clusters and survival status (OS and DFS). The results suggested that AS clusters could be used to identify patient subgroups with different survival outcomes (Fig. 6f, g), among which C1 was both associated with good outcome in OS (C1 vs. C2, *P*-value < 0.0001; C1 vs. C3, *P*-value < 0.0001) and DFS (C1 vs. C2, *P*-value = 0.0159; C1 vs. C3, *P*-value = 0.0338) analysis, followed by C3 and C2. The overall median survival for clusters (C1–C3) was 31.5 months, 7.16 months, and 14.13 months, respectively.

**Fig. 7:**
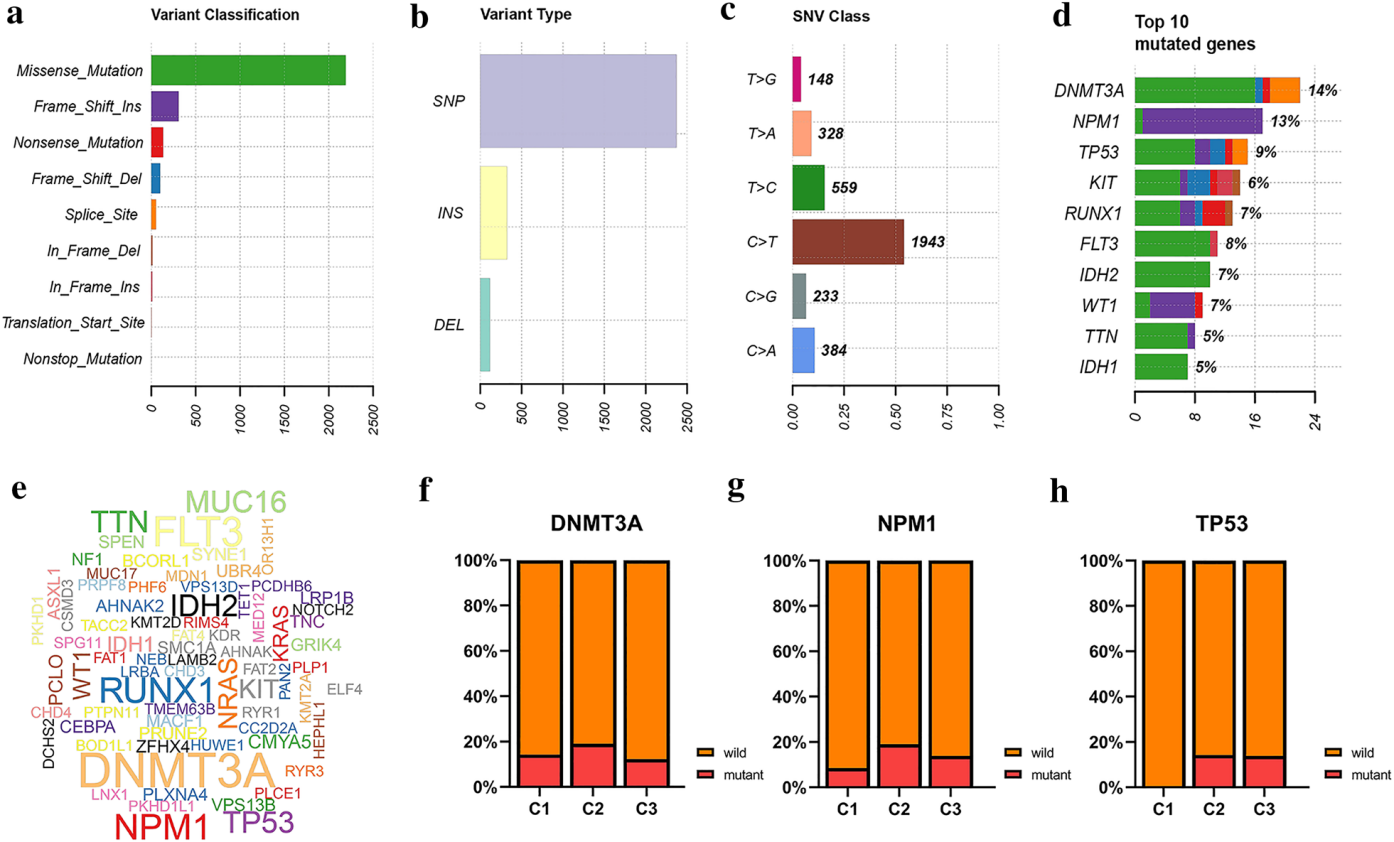
Summary of the mutation information with AML in TCGA cohort. **a** Missense mutations accounted for the largest proportion, followed by frame shift mutation and nonsense mutation. **b** Insertion or deletion occurred less frequently than single nucleotide polymorphism. **c** The C > T transversions are the predominant of single nucleotide variants (SNVs). **d**) The top 10 mutated genes in AML. **e** Cloud plots of the most frequently mutated genes. **f–h** The distribution of common mutated genes between three clusters

To further reveal the molecular characteristics of samples with AML in TCGA cohort, we performed a comprehensive molecular analysis of the mutation pattern SNPs, mutation information of each gene was exhibited according to different classified categories. Among all variant classification, missense mutations accounted for the largest proportion, followed by frame shift mutation and nonsense mutation (Fig. 7a); insertion or deletion occurred less frequently than single nucleotide polymorphism (Fig. 7b). The C > T transversions was the predominant of single nucleotide variants (SNVs) in AML (Fig. 7c). The most frequently mutated genes were exhibited in Fig. 7d, e, and the top ten includes *DNMT3A*, *NPM1*, *TP53*, *KIT*, *RUNX1*, *FLT3*, *IDH2*, *WT1*, *TTN*, and *IDH1*. Based on the AS events clustering, we found that the distribution of common mutated genes was inconsistent in different clusters (Additional file 8), the mutation frequency of DNMT3A in cluster C1-C3 was 10%, 13.8% and 11.2%, respectively (Fig. 7f); and NPM1 was 5%, 13.4% and 10.1%, respectively (Fig. 7g). Notably, no TP53 mutations were detected in C1 (Fig. 7h), which might be related to the good prognosis of C1 or the insufficient number of TP53 mutations samples. These results indicate that distinct patterns of AS are associated with different molecular characteristics.

**Fig. 8:**
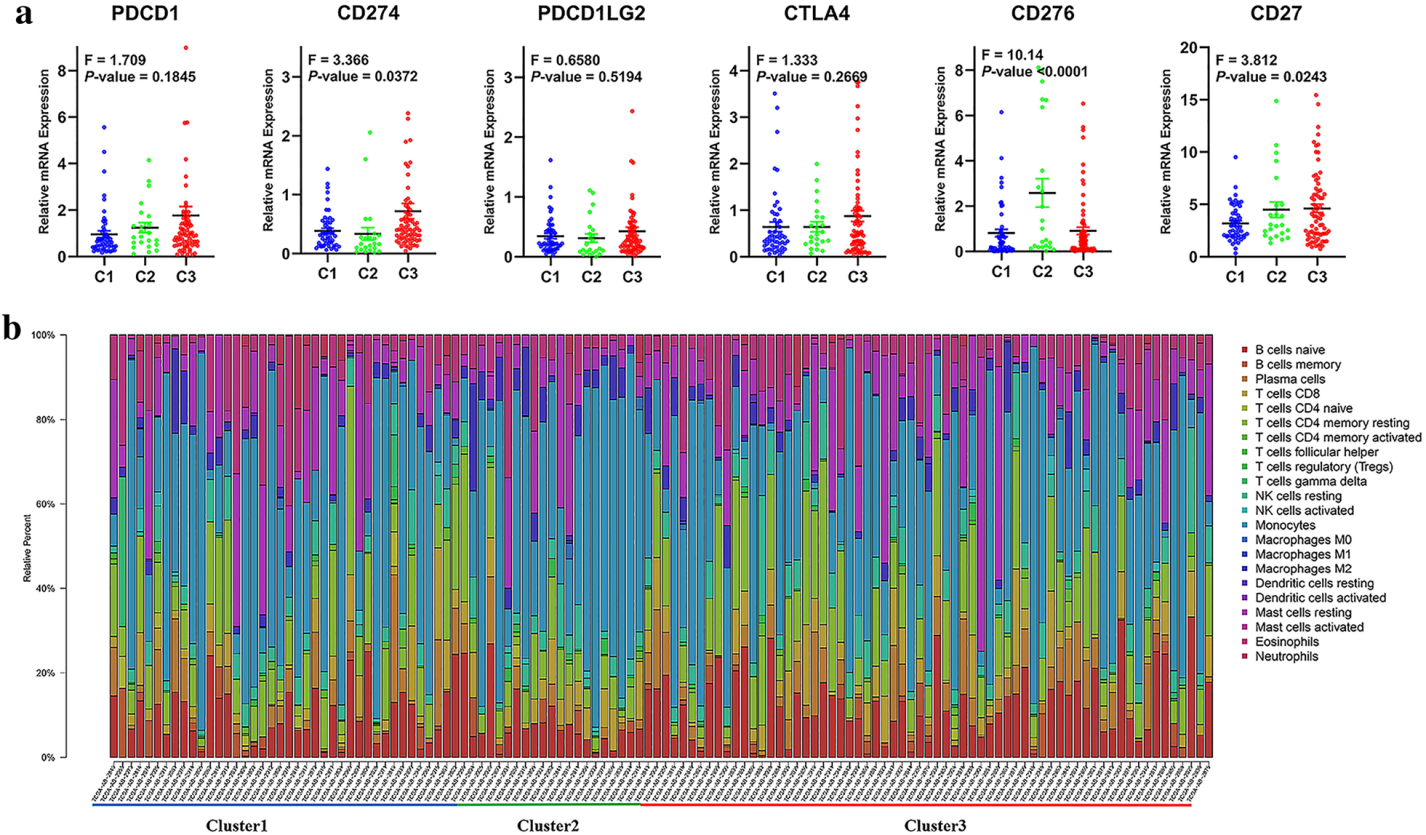
Correlation between AS-based and immune features. **a** The relationship between the expression of immune checkpoints in three clusters, including *PDCD1*, *CD274*, *PDCD1LG2*, *CTLA4*, *CD276*, and *CD27*. **b** A bar chart to illustrate the distribution of 22 immune cells in each sample

Immune checkpoint inhibitors are considered a promising treatment strategy in AML [23]. We investigated the relationship between the expression of immune checkpoints and AS clusters. As shown in Fig. 8a, *CD279*, *CD276*, *CD27* were significant different in expression distribution among the three clusters of samples. To explore the immune characteristics in bone marrow microenvironment, we generated a bar chart to illustrate the distribution of 22 immune cells in each sample by CIBERSORT algorithm (Fig. 8b). The results revealed that the most representative cell composition in bone marrow microenvironment of AML patients were monocytes, T cells CD4 memory resting, mast cells resting, B cells naive, and eosinophils. We observed that the composition of mast cells resting and B cells naive were slightly higher in C2 than C1 or C3. Collectively, these findings suggested that AML displayed distinct patterns of survival-associated AS events, and splicing events are ubiquitous and influence clinical outcome. Our findings provide new insight into molecular targeted therapy and immunotherapy strategy for AML.

## Discussion

AML is the most common hematologic malignancy, with multiple molecular subtypes and cellular heterogeneity [24]. Over the last decade, significant efforts have been made to elucidate the molecular changes in genome-wide profiling involved in AML oncogenesis. Such studies have contributed to the determination of relevant biomarkers and even therapeutic targets, including protein coding genes, microRNAs and long non-coding RNAs [25–27]. In addition, as a crucial post-transcriptional regulatory mechanism in expanding the coding capacities of genomes and increasing the diversity of proteins, AS events have been shown to have the potential in predicting clinical outcomes.

**Fig. 9:**
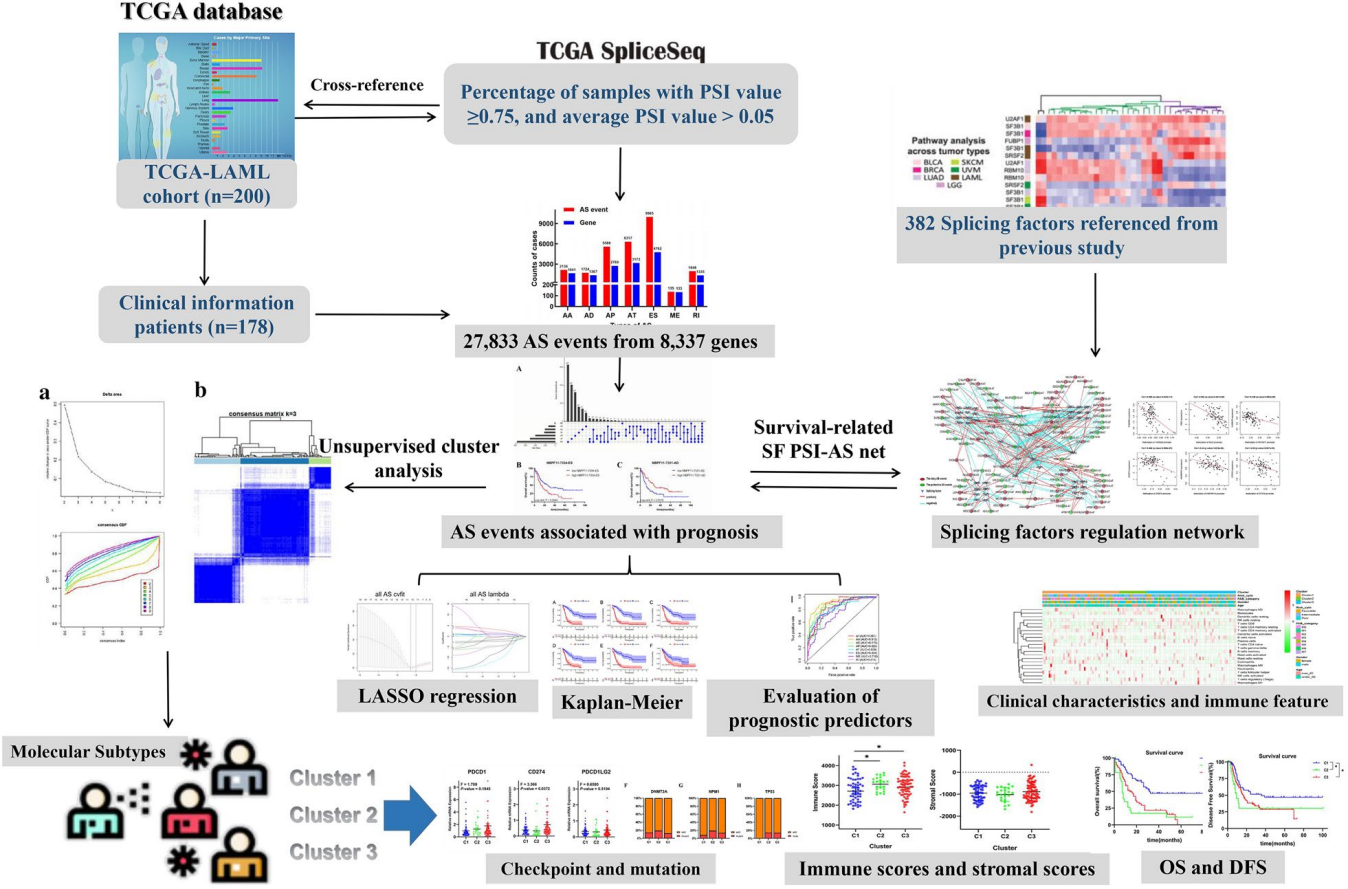
Flowchart of the systematic profiling of alternative splicing in the study design

Our whole workflow is shown in Fig. 9, this study revealed a comprehensive landscape of AS events and explored the relationship between splicing patterns and the prognosis of AML patients. A total of 27,833 AS events were detected in 8337 genes, of which ES events were the predominant type, accounting for more than 1/3 of the total AS events. Subsequently, we performed the univariate analysis for OS to evaluate the prognostic impact, and found that approximately 11% of AS events were significantly related to the clinical outcomes in AML. We found that several AS events from the same parent gene had the opposite prognosis effects. Among the survival-related AS events, AA was found to have the optimal performance in the prediction models with high classification efficiencies. AS event changes have been widely recognized in tumors that affect the protein interaction networks regulated by SFs [22, 28]. We constructed SF correlation networks to elucidate the potential mechanism of splicing pathway, and found that SFs influence disease progression by regulating the AS of downstream target genes simultaneously. Moreover, we calculated microenvironment scores by ESTIMATE algorithm. The results showed that a high immune score was an unfavorable prognostic factor for AML, and younger groups or favorable cytogenetic subtypes had lower immune scores. This is consistent with the findings of current research and supports the reliability of our results [29]. In addition, we also determined the association of AS events with immune/stromal scores to reveal the pathways involved in low- and high-immune groups. More importantly, due to the biologic and genetic heterogeneity of AML, three AS-based clusters were identified (C1–C3). Interestingly, we found that the AS-based clusters presented distinct immune features, and the outcome of survival changed accordingly, just as the C1 subgroups with low immune scores having poor clinical outcome (OS and EFS). To further reveal the association between molecular characteristics and AS-based clusters, we performed a comprehensive analysis of the mutation pattern SNPs, and investigated the relationship between the expression of immune checkpoints and AS clusters, which may be helpful in determining the ideal candidates for treatment and the optimal immunotherapy strategy.

At present, a growing number of researchers are working to develop therapies for AS. Neoantigens derived from AS have greatly expanded the pool of tumor-specific target antigens [30]. Although the identification, screening and clinical use of neoantigens still face various challenges, neoantigens with tumor tissue specificity and high immunogenicity are expected to benefit more patients as potential targets for cellular immunotherapy [31, 32]. Previously, TCGA as the principal guide to understanding of the complex tumor biology [33], large-scale sequencing data were used to identify prognostic AS events and revealed pathogenesis through regulatory splicing networks in colorectal cancer and head-neck carcinoma [34, 35]. More and more AS-related prognostic models have been established, showing excellent efficacy in various cancers. Researcher have used splicing changes to construct predictive models for different types of tumors, such as hepatocellular carcinoma, glioblastoma, cervical cancer, sarcoma, and esophageal carcinoma [36–40]. Interestingly, AML has been identified to share some common prognostic markers of AS with other tumors. Chen et al. demonstrated the survival-related AS events based on TCGA database, and they provided an overview of misregulated AS events in different types of AML [41]. In the meantime, Jin et al. reported that the AUC of ROC curve for the final prediction model constructed with 15 AS events was 0.931, which might refine risk stratification of the European Leukemia Net (ELN) [42]. But they did not explore the association between the AS events and the clinical characteristics of AML patients. This work was actually greatly increases the confidence in this important breakthrough [43]. Another argument for AS events was critical to improve the understanding of tumor resistance as the destruction of the activity of the splicing factor SRSF3 could lead to drug resistance in the immunotherapy of leukemia [44]. Although a large number of tumor-specific AS events can be obtained through transcriptome data analysis, not all splicing events can be translated into proteins. Further analysis is required to determine the presence of splicing isomers by protein spectroscopy. Currently, there are two application strategies for AS events as a therapeutic target, one being to design an antisense nucleotide for specific AS events to restore the phenotype of normal cells [12, 45], and the other being to apply small-molecular compound to regulate SFs [46]. However, splicing itself can also be used as a direct drug target, *MET* exon14-skipping have been found in non-small-cell lung cancer patients who are sensitive to *MET* targeted therapy [47]. Moreover, these therapeutic strategies may have different effects depending on the tumor type and SFs expression or mutation [14, 22]. Our study reflects some manifestations of AML, we have highlighted focused on subtype clustering of AML patients with AS events. For adoptive immunotherapy, the tumor specificity of antigen and cross-reactivity of T cell receptor are the two most important factors affecting clinical safety. Therefore, targeted therapies based on AS may bring new hopes when existing targeted drugs fail.

## Conclusions

Our study analyzed the landscape of AS events and identified their association with clinical outcome. Our findings support the notion that splicing events are ubiquitous in AML patient. SFs correlation networks further altered the diversity of AS events through epigenetic regulation and clarified the potential mechanism of the splicing pathway. Moreover, clustering based on prognostic AS signature revealed the clinical characteristics of bone marrow immune microenvironment. This discovery provided new insight into molecular targeted therapy and immunotherapy strategy for AML.

## Methods

### Data curation process

RNA sequencing profiles and corresponding clinical information were available at TCGA portal data (https://portal.gdc.cancer.gov/). Meanwhile, we evaluated the RNA splicing patterns from 178 AML patients using SpliceSeq software (https://bioinformatics.mdanderson.org/TCGASpliceSeq/) and generated the AS profiles of genes for each patient [48]. The ratio between reads including or excluding exons, also known as Percent Spliced In (PSI) value, was calculated for each detected AS events [49]. Seven different types of splice events were identified including Alternate Acceptor site (AA), Alternate Donor site (AD), Alternate Promoter (AP), Alternate Terminator (AT), Exon Skip (ES), Mutually Exclusive Exons (ME), and Retained Intron (RI). We set strict selection criteria (Percentage of samples with PSI value ≥ 0.75, and average PSI value > 0.05) to generate a reliable set of AS events. To describe an AS event precisely, every AS event was assigned a unique annotation with gene symbol, the ID number from SpliceSeq database and splicing pattern.

### Prognostic signatures for alternative splicing events

We used Perl language (https://www.perl.org/; version 5.30.0) to extract the matrix file and match array with clinical follow-up data. Univariate Cox regression analysis was performed to screen prognostic related AS events in AML. The z-score is positive if the value lies above the mean, and negative if it lies below the mean [50], and bubble plots were used to visualize data. The interactive sets between seven types of AS were drawn by the ‘UpsetR’ package in R 3.5.2 software (https://www.r-project.org/). The major codes used in this study could be found in the Additional file 8. The least absolute shrinkage and selection operator (LASSO) regression were performed to screen the most significant AS events, and all variables left after elimination are considered selected [51]. We performed the penalty parameter lambda by the cross-validation using the R package ‘glmnet’. The optimal lambda value corresponding to the minimum value of the cross-validation error mean was screened to determine the potential survival-related AS events (Additional file 9: Figure S3). The optimal survival-related AS events in each AS types were used to constructed predictive models by using multivariate cox regression. Furthermore, AML patients were divided into two groups by using the median risk score, the formula is as follows: RiskScore = PSI of AS1*β1AS1 + PSI of AS2*β2AS2 +…PSI of ASn*βnASn. Kaplan–Meier (K-M) survival analysis, receiver operating characteristic (ROC) curves and the area under the curve (AUC) were used to assess the predictive accuracy for each prognostic signature. The *P*-value was computed using log-rank test.

### Splicing factor regulated network construction

A list of 382 splicing factors(SF) genes were referenced from a previous study and relevant databases [52, 53]. The expression profiles of SFs were obtained from TCGA and normalized by using all housekeeping genes with log base 2 transformed. Pearson correlation test was used to evaluate the correlation between SFs expression and PSI values of survival-associated AS events, and *P*-values were adjusted by Benjamini & Hochberg (BH) correlation (*P*-value < 0.05, |Pearson’s coefficient| > 0.65). Cytoscape (version 3.7.1, http://www.cytoscape.org/) is a helpful tool for visualizing molecular interaction network and observing the correlation between molecules, we used to plot the AS regulatory network. The methylation beta values of samples measured by the infinium human methylation 450 K platform were downloaded from TCGA portal. Moreover, correlation analysis was performed between SFs methylation and SFs mRNA expression, and the copy number variation (CNV) of SFs was calculated in AML.

### Immune characteristics analysis

The immune and stromal scores of each sample were calculated by applying the ESTIMATE algorithm [54] to assess bone marrow immune activity, and AML cases were divided into low- and high-score groups according to the median of immune/stromal scores. Gene set enrichment analysis (GSEA, version 4.0.1, http://software.broadinstitute.org/gsea/) is a knowledge-based method which determines whether a particular set of functionally related genes shows statistically significance, and we used it to verify the differences in molecular pathways between low- and high-immune score groups [55]. The ‘c2.cp.kegg.v7.1.symbols.gmt’ was selected as the database of gene-sets and 1000 permutations were performed to generate the null distributions. In order to evaluate the bone marrow microenvironment in AML patients, we estimated the abundance of various types of immune cell by using the CIBERSORT algorithm [56], which is a versatile computational method for quantifying 22 immune cell types from bulk tissue transcriptomes. Each sample in the data set were performed 100 times for further study, and samples with a *P*-value < 0.05 was set as the cutoff.

### Evaluation of the correlation with clinical features

To visualize the associations between survival-associated AS events and the heterogeneity of AML patients, we clustered the AML into different groups with hierarchical consensus clustering by using the R package ‘Consensus Cluster Plus’ [57]. In our study, the cumulative distribution function (CDF) curve was used to determine the best number of clusters, parameters were set as default except that max K was set at 9. We selected three sub-types (k = 3, C1–C3) that optimally fit the data. The association between clinical variables and sub-types, including gender, age, cytogenetics risk category, French American British (FAB) subtype, and bone marrow immune features, was analyzed. Kaplan–Meier curves and log-rank test were used to compare the overall survival (OS) and disease free survival (DFS) of different subtypes. Moreover, single nucleotide polymorphism (SNP) and molecular alteration of AML were described by using R package ‘maftools’ (version 2.2.10). Molecules with high mutation frequencies (DNMT3A, NPM1, and TP53) were included in the evaluation subtype.

### Statistical analyses

GraphPad Prism (version 8.02) and R software (version 3.5.2) were used for analysis. Pearson’ s correlation test and spearman’ s correlation test were employed in the correlation analysis. The Student’s t-test was utilized to compare the differences in variables between groups, and *P*-value less than 0.05 were deemed statistically significant.

## Supplementary information

**Additional file 1: Table S1**. Clinical characteristics of patients with AML.

**Additional file 2: Table S2**. The detailed information and prognostic signature of the detected AS events in AML.

**Additional file 3: Figure S1.** Upset plot and survival diagram for seven types of AS events in AML. **a** Upset plot of parent gene interactions between the seven types of survival-associated AS events. **b-e** Representative Kaplan–Meier curves for OS according to PSI value of AS events of a parent gene showing the opposite prognosis. Depicted P-values are from log-rank tests.

**Additional file 4: Figure S2.** Kaplan–Meier plots and ROC curves of prognostic predictors for AML patients. **a-h** Kaplan–Meier plots of prediction models associated with OS constructed with AA, AD, AP, AT, ES, ME, RI events and all types of AS events, respectively. **i** The ROC curves with AUCs of the predictive models for each AS markers.

**Additional file 5: Table S3**. Details of prediction models for acute myelocytic leukemia based on each type of splicing event.

**Additional file 6: Table S4**. Correlation between splicing factor and survival-associated alternative splicing events in AML.

**Additional file 7: Table S5**. Correlation between splicing factor (SF) expression and SF methylation in AML.

**Additional file 8:**. Major R codes and some indirect results

**Additional file 9: Figure S3.** Selection of the optimal survival-related AS events used for construction of the final prediction model by LASSO regression. (A, C, E, G, I, K, M, O) Dotted vertical lines were drawn at the optimal values by using the minimum criteria. (B, D, F, H, J, L, N, P) LASSO coefficient profiles of the candidate survival-related AS events.

## Data Availability

The gene expression profiles and clinical data can be found at the GDC portal (https://portal.gdc.cancer.gov/). Software and resources used for the analyses are described in each method section. All results generated in this study can be found in supplementary tables.

## Abbreviations

AS: Alternative splicing
AML: Acute myeloid leukemia
PSI: Percent Spliced In
SF: Splicing factor
HR: Hazard ratio
ROC: Receiver operating characteristic
OS: Overall survival
DFS: Disease free survival
AA: Alternate Acceptor site
AD: Alternate Donor site
AP: Alternate Promoter
AT: Alternate Terminator
ES: Exon Skip
ME: Mutually Exclusive Exons
RI: Retained Intron
CNV: Copy number variation
SNP: Single nucleotide polymorphism

## References

[1] Gambacorta V, Gnani D, Vago L, Di Micco R (2019). Epigenetic therapies for acute myeloid leukemia and their immune-related effects. Front Cell Dev Biol.

[2] Sami SA, Darwish NHE, Barile ANM, Mousa SA (2020). Current and future molecular targets for acute myeloid leukemia therapy. Curr Treat Options Oncol.

[3] Siegel RL, Miller KD, Jemal A (2020). Cancer statistics, 2020. CA Cancer J Clin.

[4] Lee CJ, Labopin M, Beelen D, Finke J, Blaise D, Ganser A (2019). Comparative outcomes of myeloablative and reduced-intensity conditioning allogeneic hematopoietic cell transplantation for therapy-related acute myeloid leukemia with prior solid tumor: a report from the acute leukemia working party of the European society for blood and bone marrow transplantation. Am J Hematol.

[5] Kumar B, Garcia M, Weng L, Jung X, Murakami JL, Hu X (2018). Acute myeloid leukemia transforms the bone marrow niche into a leukemia-permissive microenvironment through exosome secretion. Leukemia.

[6] Batsivari A, Haltalli MLR, Passaro D, Pospori C, Lo Celso C, Bonnet D (2020). Dynamic responses of the haematopoietic stem cell niche to diverse stresses. Nat Cell Biol.

[7] Charrot S, Armes H, Rio-Machin A, Fitzgibbon J (2020). AML through the prism of molecular genetics. Br J Haematol.

[8] Carbonell D, Suárez-González J, Chicano M, Andrés-Zayas C, Triviño JC, Rodríguez-Macías G, et al. Next-Generation Sequencing Improves Diagnosis, Prognosis and Clinical Management of Myeloid Neoplasms. Cancers (Basel) 2019. 11. 10.3390/cancers11091364. 10.3390/cancers11091364 PMC6770229 31540291

[9] Nilsen TW, Graveley BR (2010). Expansion of the eukaryotic proteome by alternative splicing. Nature.

[10] Trincado JL, Sebestyén E, Pagés A, Eyras E (2016). The prognostic potential of alternative transcript isoforms across human tumors. Genome Med.

[11] Ghigna C, Giordano S, Shen H, Benvenuto F, Castiglioni F, Comoglio PM (2005). Cell motility is controlled by SF2/ASF through alternative splicing of the Ron protooncogene. Mol Cell.

[12] Bechara EG, Sebestyén E, Bernardis I, Eyras E, Valcárcel J (2013). RBM5, 6, and 10 differentially regulate NUMB alternative splicing to control cancer cell proliferation. Mol Cell.

[13] Anczuków O, Krainer AR (2016). Splicing-factor alterations in cancers. RNA.

[14] Dvinge H, Kim E, Abdel-Wahab O, Bradley RK (2016). RNA splicing factors as oncoproteins and tumour suppressors. Nat Rev Cancer.

[15] Comoli P, Chabannon C, Koehl U, Lanza F, Urbano-Ispizua A, Hudecek M (2019). Development of adaptive immune effector therapies in solid tumors. Ann Oncol.

[16] Hegde PS, Chen DS (2020). Top 10 Challenges in Cancer Immunotherapy. Immunity.

[17] Mimura N, Hideshima T, Anderson KC (2015). Novel therapeutic strategies for multiple myeloma. Exp Hematol.

[18] Jewer M, Findlay SD, Postovit L-M (2012). Post-transcriptional regulation in cancer progression : microenvironmental control of alternative splicing and translation. J Cell Commun Signal.

[19] Sebestyén E, Zawisza M, Eyras E (2015). Detection of recurrent alternative splicing switches in tumor samples reveals novel signatures of cancer. Nucleic Acids Res.

[20] Shen S, Wang Y, Wang C, Wu YN, Xing Y (2016). SURVIV for survival analysis of mRNA isoform variation. Nat Commun.

[21] Trapnell C, Pachter L, Salzberg SL (2009). TopHat: discovering splice junctions with RNA-Seq. Bioinformatics.

[22] Shukla GC, Singh J (2017). Mutations of RNA splicing factors in hematological malignancies. Cancer Lett.

[23] Ghosh A, Barba P, Perales M (2020). Checkpoint inhibitors in AML: are we there yet?. Br J Haematol.

[24] Wiggers CRM, Baak ML, Sonneveld E, Nieuwenhuis EES, Bartels M, Creyghton MP (2019). AML subtype is a major determinant of the association between prognostic gene expression signatures and their clinical significance. Cell Rep.

[25] Zhang N, Chen Y, Shen Y, Lou S, Deng J (2019). Comprehensive analysis the potential biomarkers for the high-risk of childhood acute myeloid leukemia based on a competing endogenous RNA network. Blood Cells Mol Dis.

[26] Liu Y, Cheng Z, Pang Y, Cui L, Qian T, Quan L (2019). Role of microRNAs, circRNAs and long noncoding RNAs in acute myeloid leukemia. J Hematol Oncol.

[27] Li M, Cui X, Guan H (2020). MicroRNAs: pivotal regulators in acute myeloid leukemia. Ann Hematol.

[28] Ellis JD, Barrios-Rodiles M, Colak R, Irimia M, Kim T, Calarco JA (2012). Tissue-specific alternative splicing remodels protein-protein interaction networks. Mol Cell.

[29] Yan H, Qu J, Cao W, Liu Y, Zheng G, Zhang E (2019). Identification of prognostic genes in the acute myeloid leukemia immune microenvironment based on TCGA data analysis. Cancer Immunol Immunother.

[30] Kahles A, Lehmann K-V, Toussaint NC, Hüser M, Stark SG, Sachsenberg T (2018). Comprehensive analysis of alternative splicing across tumors from 8,705 patients. Cancer Cell.

[31] Frankiw L, Baltimore D, Li G (2019). Alternative mRNA splicing in cancer immunotherapy. Nat Rev Immunol.

[32] Ott PA, Hu Z, Keskin DB, Shukla SA, Sun J, Bozym DJ (2017). An immunogenic personal neoantigen vaccine for patients with melanoma. Nature.

[33] Carrot-Zhang J, Chambwe N, Damrauer JS, Knijnenburg TA, Robertson AG, Yau C (2020). Comprehensive analysis of genetic ancestry and its molecular correlates in cancer. Cancer Cell.

[34] Xiong Y, Deng Y, Wang K, Zhou H, Zheng X, Si L (2018). Profiles of alternative splicing in colorectal cancer and their clinical significance: a study based on large-scale sequencing data. EBioMedicine.

[35] Li Z-X, Zheng Z-Q, Wei Z-H, Zhang L-L, Li F, Lin L (2019). Comprehensive characterization of the alternative splicing landscape in head and neck squamous cell carcinoma reveals novel events associated with tumorigenesis and the immune microenvironment. Theranostics.

[36] Yang L, He Y, Zhang Z, Wang W (2019). Systematic analysis and prediction model construction of alternative splicing events in hepatocellular carcinoma: a study on the basis of large-scale spliceseq data from The Cancer Genome Atlas. PeerJ.

[37] Chen X, Zhao C, Guo B, Zhao Z, Wang H, Fang Z (2019). Systematic profiling of alternative mRNA splicing signature for predicting glioblastoma prognosis. Front Oncol.

[38] Shao X-Y, Dong J, Zhang H, Wu Y-S, Zheng L (2020). Prognostic value and potential role of alternative mRNA splicing events in cervical cancer. Front Genet.

[39] Hu C, Wang Y, Liu C, Shen R, Chen B, Sun K, et al. Systematic Profiling of Alternative Splicing for Sarcoma Patients Reveals Novel Prognostic Biomarkers Associated with Tumor Microenvironment and Immune Cells. Med Sci Monit. 2020. 26:e924126. 10.12659/MSM.924126. 10.12659/MSM.924126 PMC7388651 32683393

[40] Sun J-R, Kong C-F, Lou Y-N, Yu R, Qu X-K, Jia L-Q (2020). Genome-wide profiling of alternative splicing signature reveals prognostic predictor for esophageal carcinoma. Front Genet.

[41] Chen X-X, Zhu J-H, Li Z-P, Xiao H-T, Zhou H (2020). Comprehensive characterization of the prognosis value of alternative splicing events in acute myeloid leukemia. DNA Cell Biol.

[42] Jin P, Tan Y, Zhang W, Li J, Wang K (2020). Prognostic alternative mRNA splicing signatures and associated splicing factors in acute myeloid leukemia. Neoplasia.

[43] Anande G, Deshpande NP, Mareschal S, Batcha AMN, Hampton HR, Herold T (2020). RNA splicing alterations induce a cellular stress response associated with poor prognosis in acute myeloid leukemia. Clin Cancer Res.

[44] Sotillo E, Barrett DM, Black KL, Bagashev A, Oldridge D, Wu G (2015). Convergence of acquired mutations and alternative splicing of CD19 enables resistance to CART-19 immunotherapy. Cancer Discov.

[45] Ghigna C, De Toledo M, Bonomi S, Valacca C, Gallo S, Apicella M (2010). Pro-metastatic splicing of Ron proto-oncogene mRNA can be reversed: therapeutic potential of bifunctional oligonucleotides and indole derivatives. RNA Biol.

[46] Salton M, Misteli T (2016). Small molecule modulators of Pre-mRNA splicing in cancer therapy. Trends Mol Med.

[47] Heist RS, Shim HS, Gingipally S, Mino-Kenudson M, Le L, Gainor JF (2016). MET Exon 14 skipping in non-small cell lung cancer. Oncologist.

[48] Ryan M, Wong WC, Brown R, Akbani R, Su X, Broom B (2016). TCGASpliceSeq a compendium of alternative mRNA splicing in cancer. Nucleic Acids Res.

[49] Ryan MC, Cleland J, Kim R, Wong WC, Weinstein JN (2012). SpliceSeq: a resource for analysis and visualization of RNA-Seq data on alternative splicing and its functional impacts. Bioinformatics.

[50] Schauer DP, Eckman MH (2014). The use of z scores in probabilistic sensitivity analyses. Med Decis Making.

[51] Gao J, Kwan PW, Shi D (2010). Sparse kernel learning with LASSO and Bayesian inference algorithm. Neural Netw.

[52] Seiler M, Peng S, Agrawal AA, Palacino J, Teng T, Zhu P (2018). Somatic mutational landscape of splicing factor genes and their functional consequences across 33 cancer types. Cell Rep.

[53] Giulietti M, Piva F, D’Antonio M, D’Onorio De Meo P, Paoletti D, Castrignanò T, et al. SpliceAid-F: a database of human splicing factors and their RNA-binding sites. Nucleic Acids Res 2013. 41:D125-131. 10.1093/nar/gks997. 10.1093/nar/gks997 PMC3531144 23118479

[54] Becht E, Giraldo NA, Lacroix L, Buttard B, Elarouci N, Petitprez F (2016). Estimating the population abundance of tissue-infiltrating immune and stromal cell populations using gene expression. Genome Biol.

[55] Subramanian A, Tamayo P, Mootha VK, Mukherjee S, Ebert BL, Gillette MA (2005). Gene set enrichment analysis: a knowledge-based approach for interpreting genome-wide expression profiles. Proc Natl Acad Sci USA.

[56] Newman AM, Liu CL, Green MR, Gentles AJ, Feng W, Xu Y (2015). Robust enumeration of cell subsets from tissue expression profiles. Nat Methods.

[57] Wilkerson MD, Hayes DN (2010). ConsensusClusterPlus: a class discovery tool with confidence assessments and item tracking. Bioinformatics.

