# Supplementary figures and images for: Clusterization in acute myeloid leukemia based on prognostic alternative splicing signature to reveal the clinical characteristics in the bone marrow microenvironment
Source: Cell Biosci. 2020 Oct 12;10:118. doi: 10.1186/s13578-020-00481-5 (PMC7552347; doi:10.1186/s13578-020-00481-5)

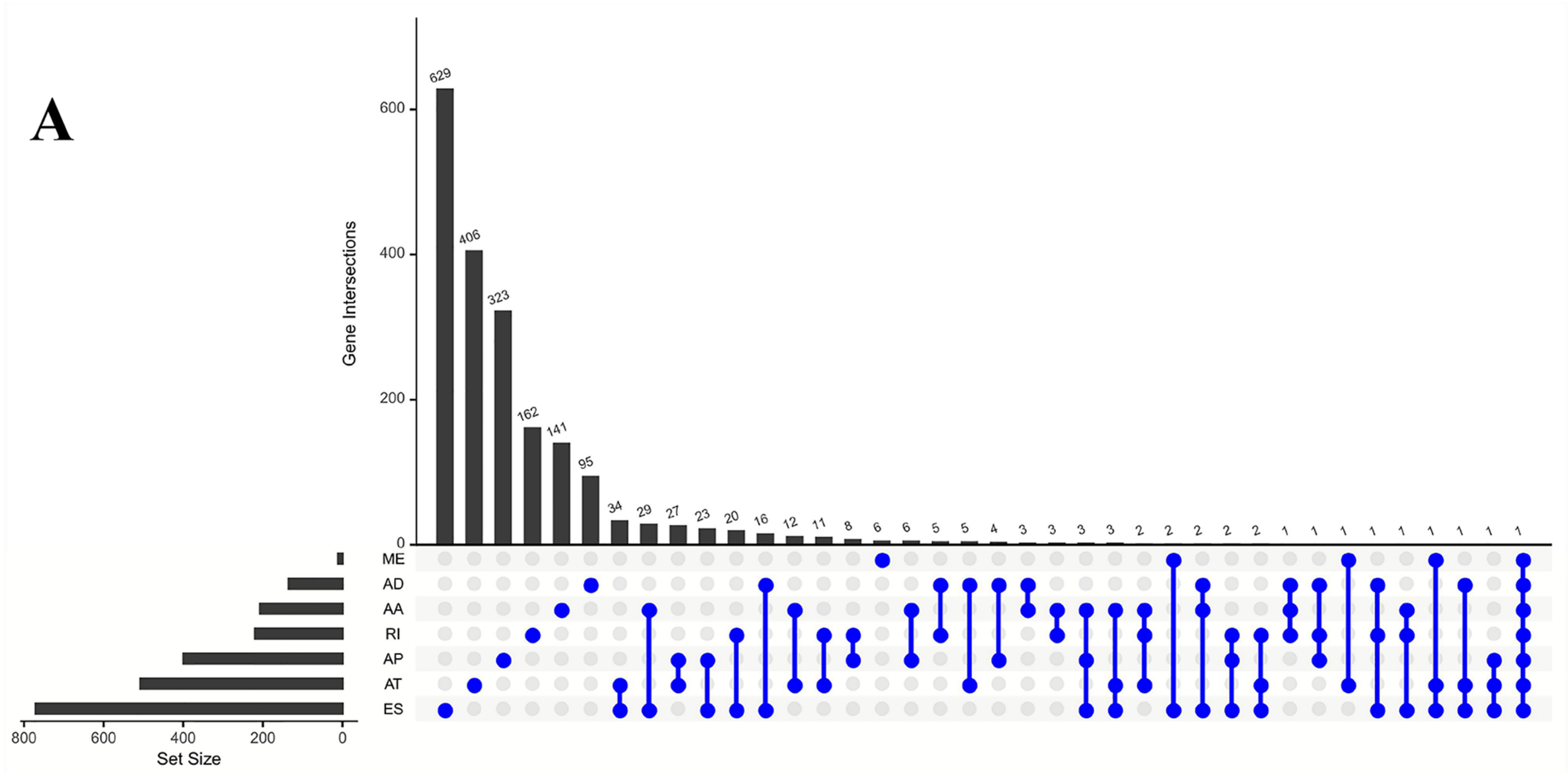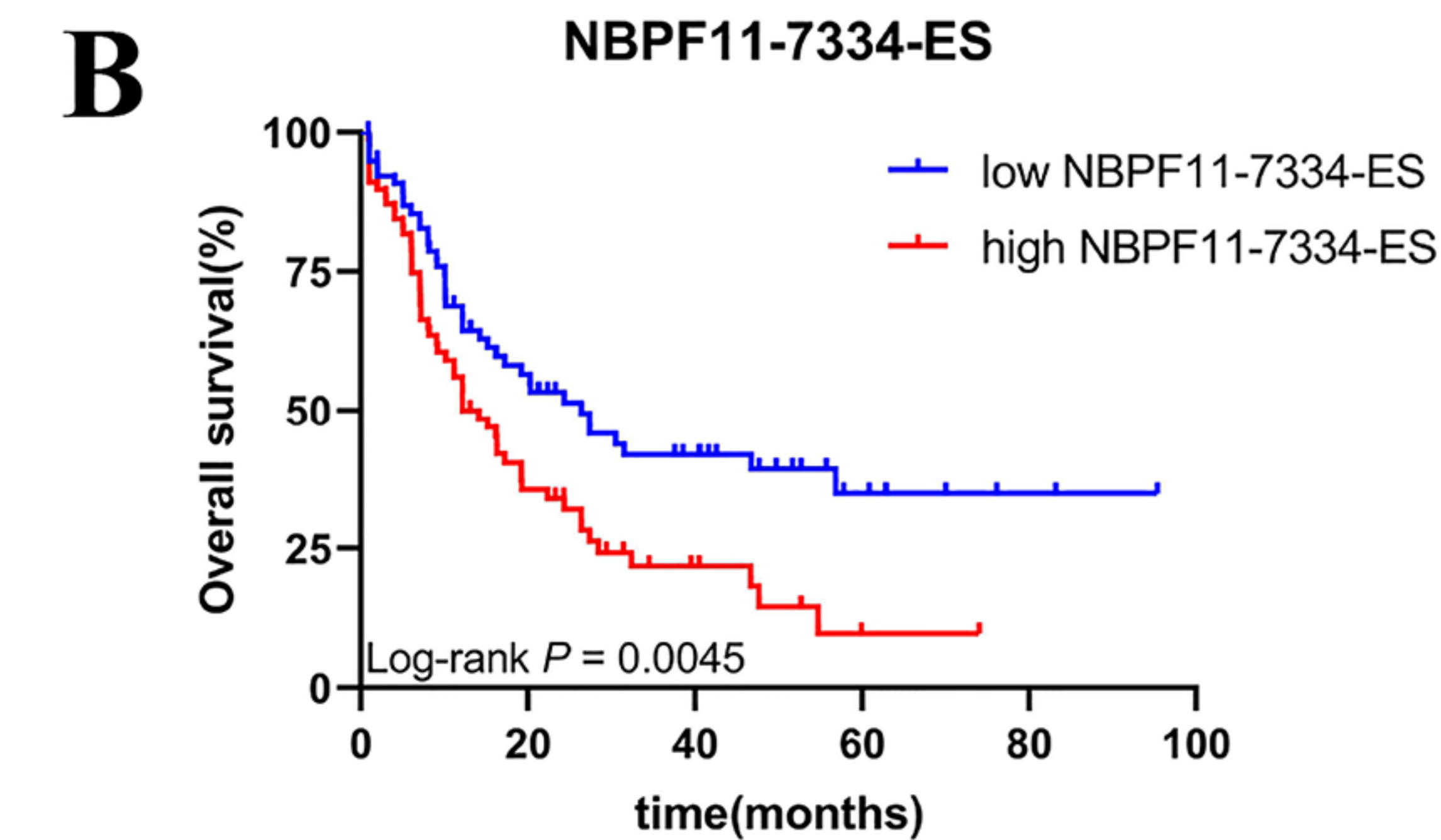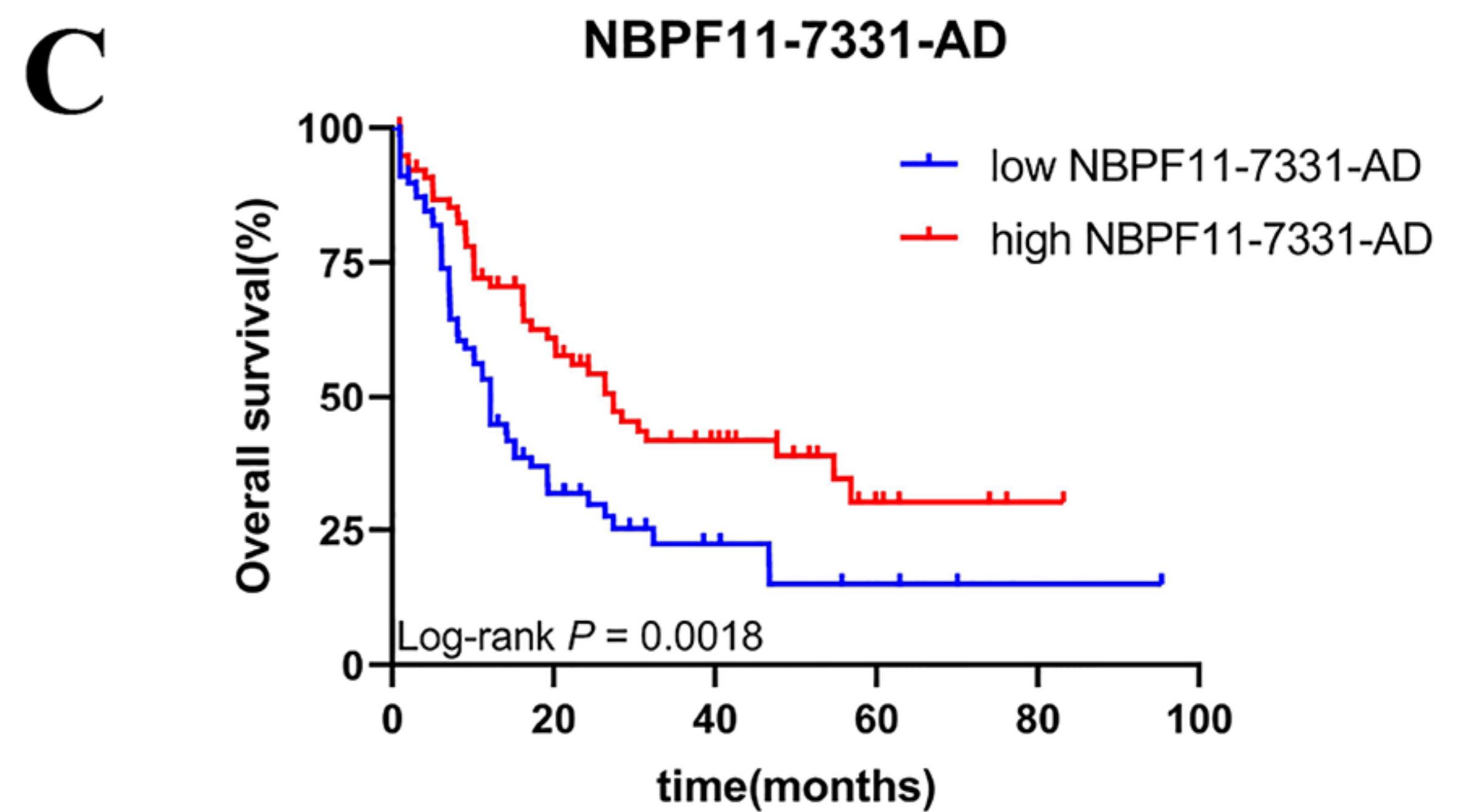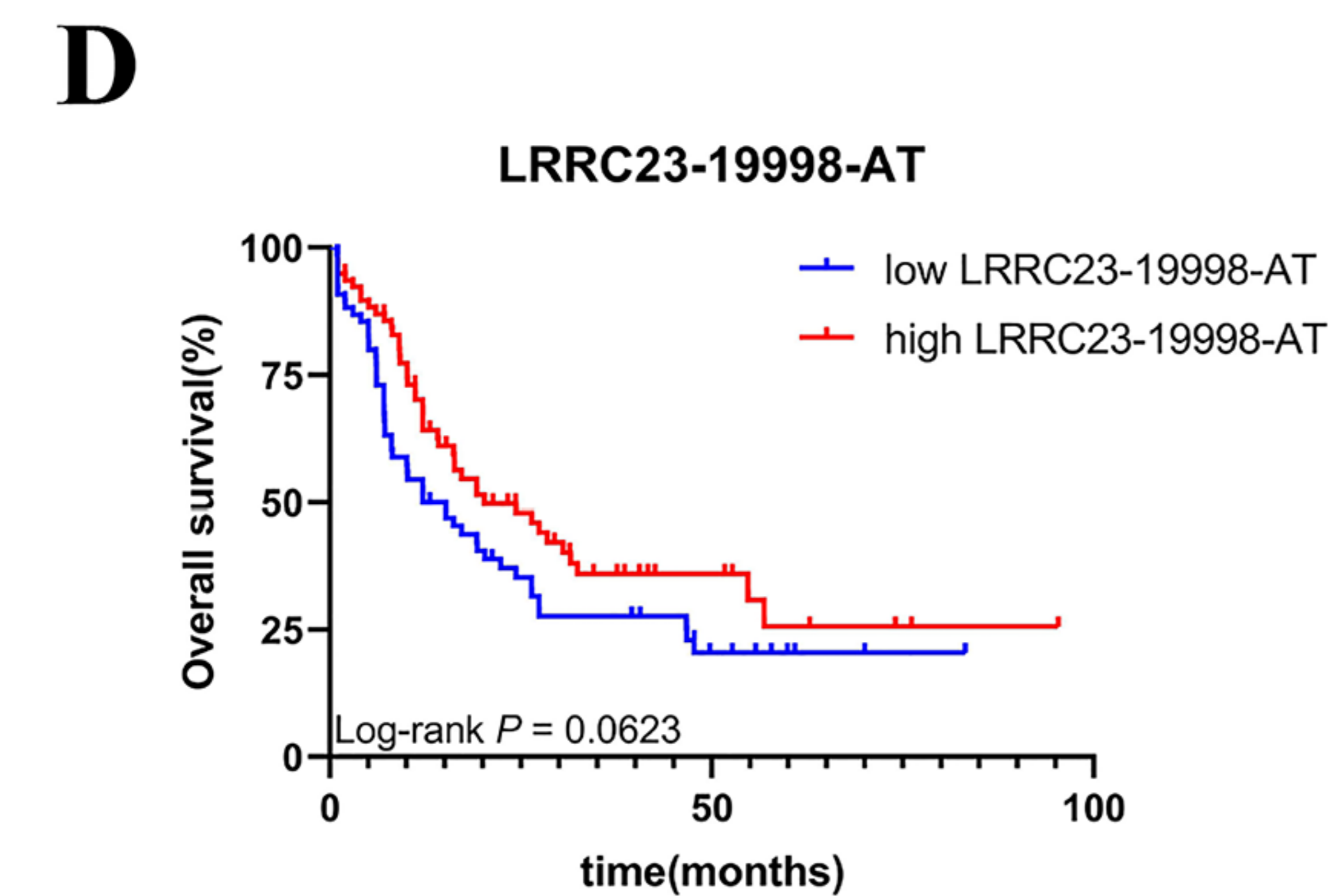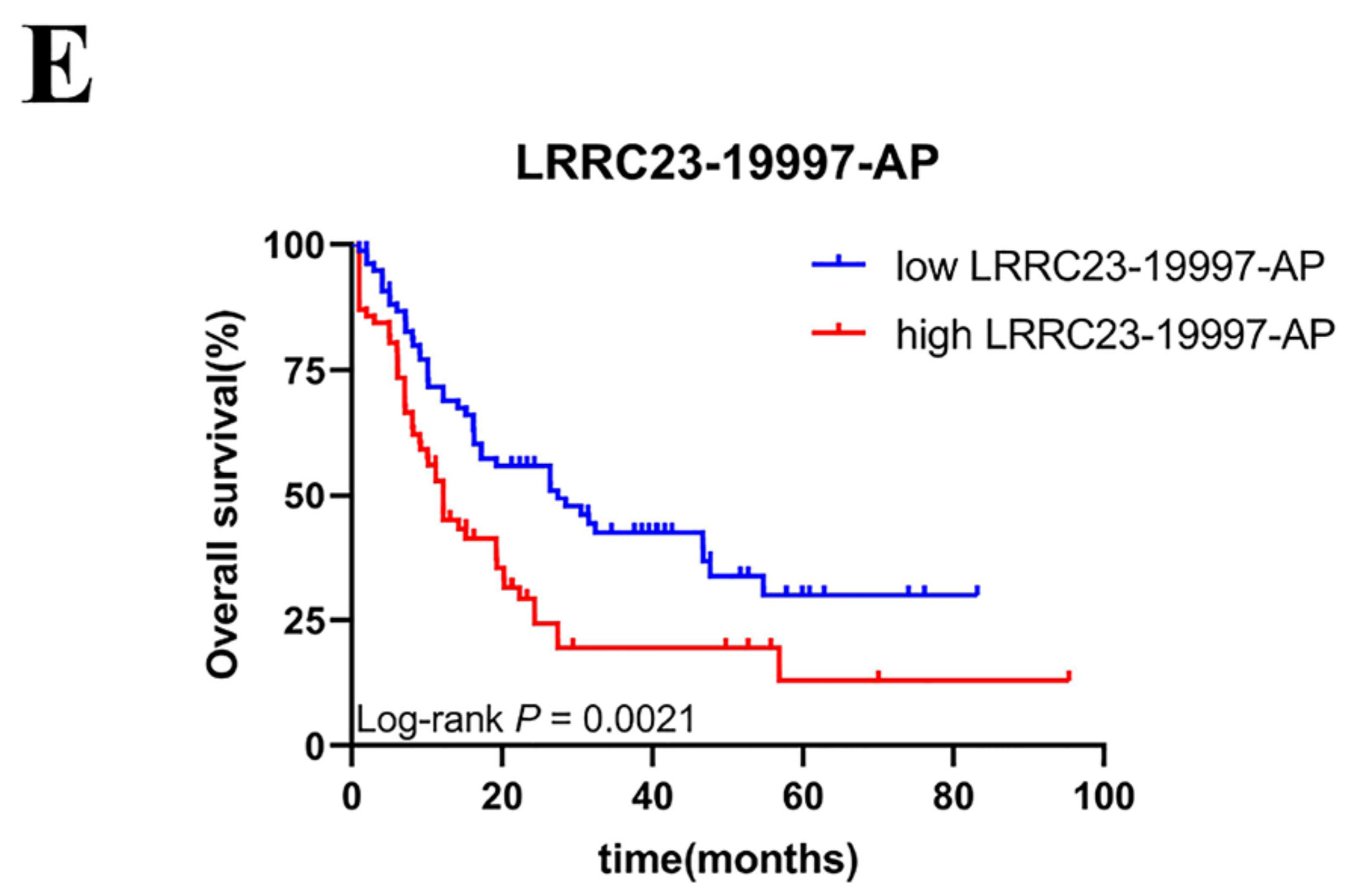

Supplement: Supplementary file 3 — Additional file 3: Figure S1. Upset plot and survival diagram for seven types of AS events in AML. a Upset plot of parent gene interactions between the seven types of survival-associated AS events. b-e Representative Kaplan–Meier curves for OS according to PSI value of AS events of a parent gene showing the opposite prognosis. Depicted P-values are from log-rank tests. [file 13578_2020_481_MOESM3_ESM.pdf]

**A**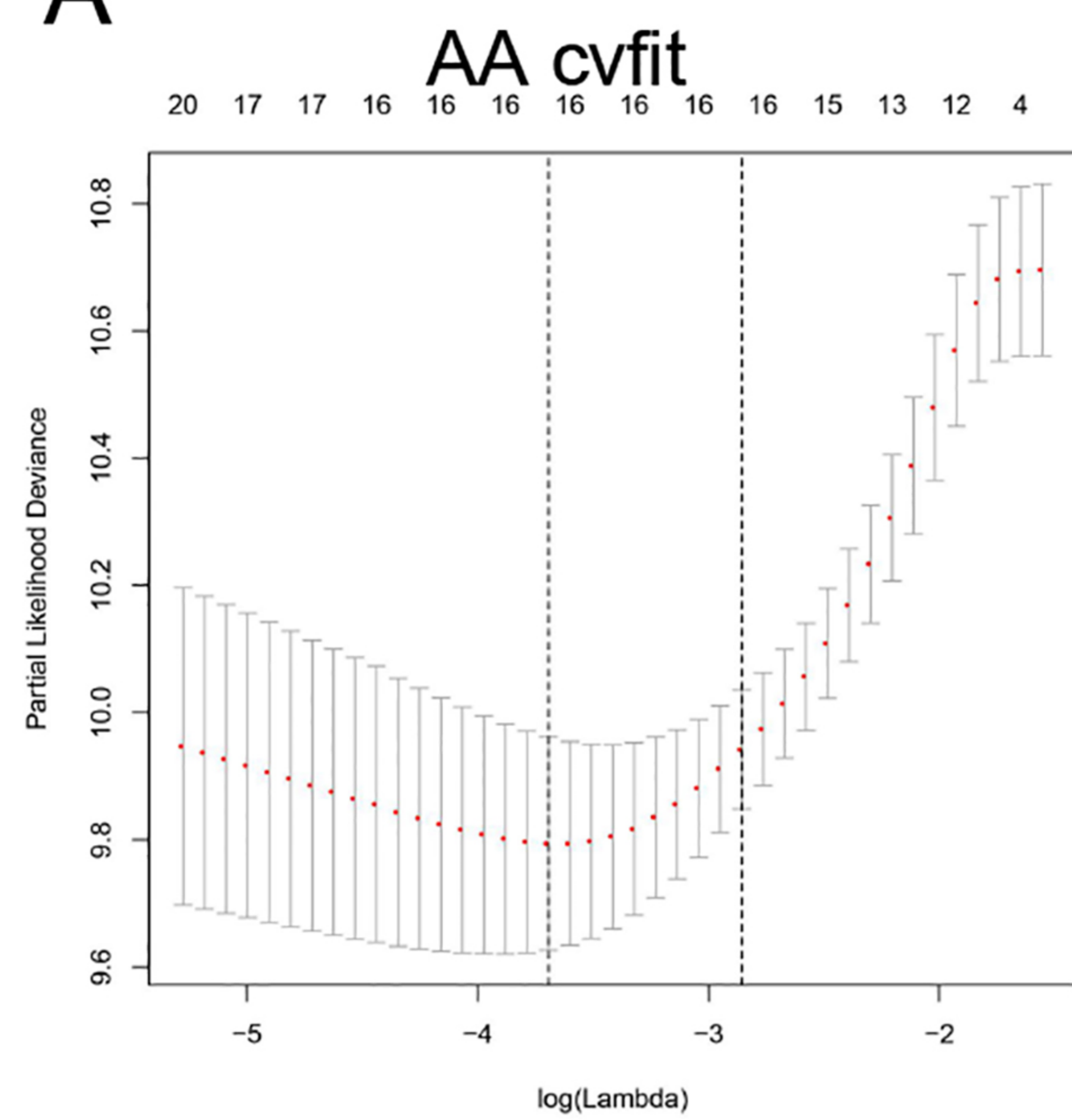**B**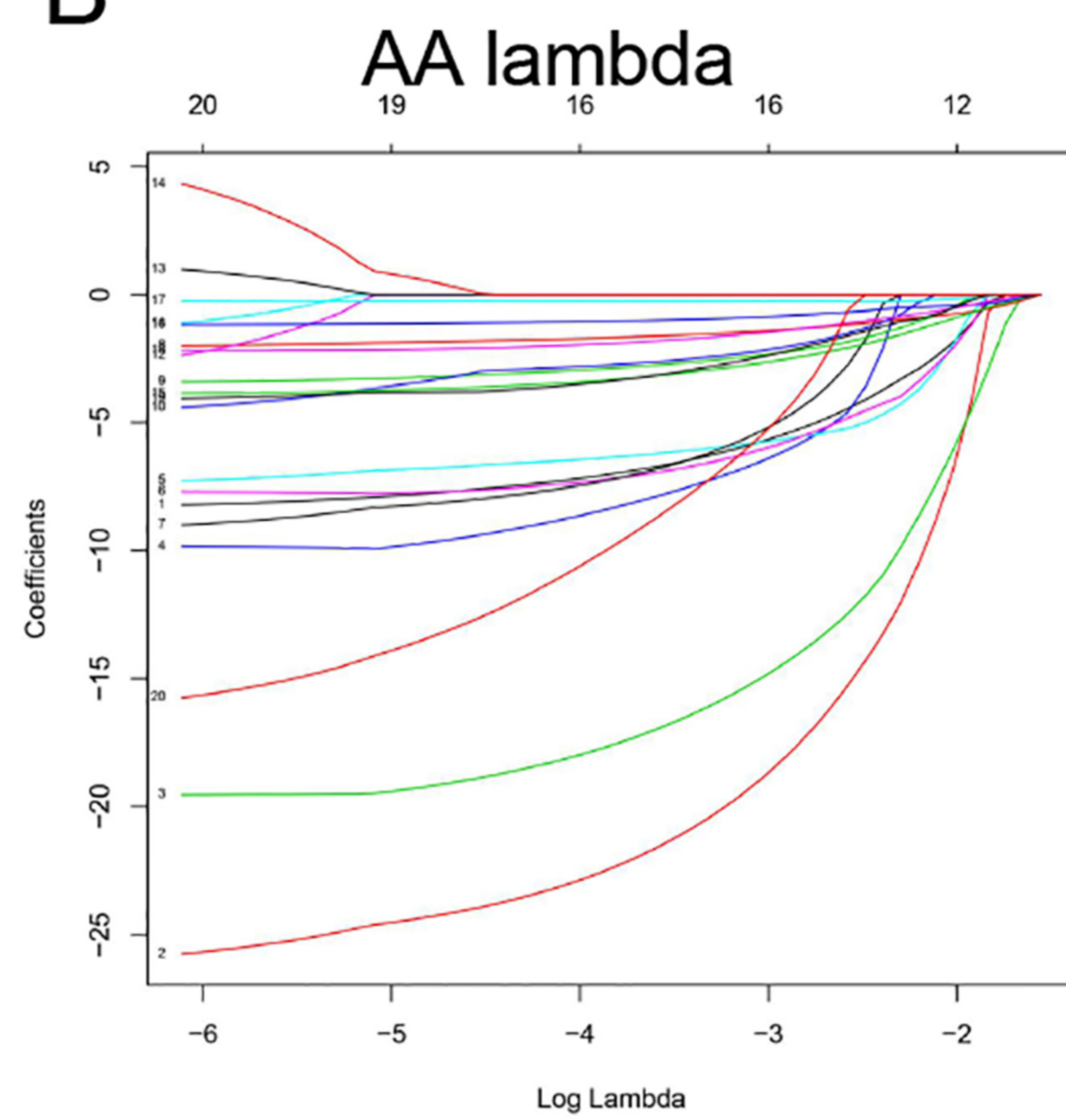**C**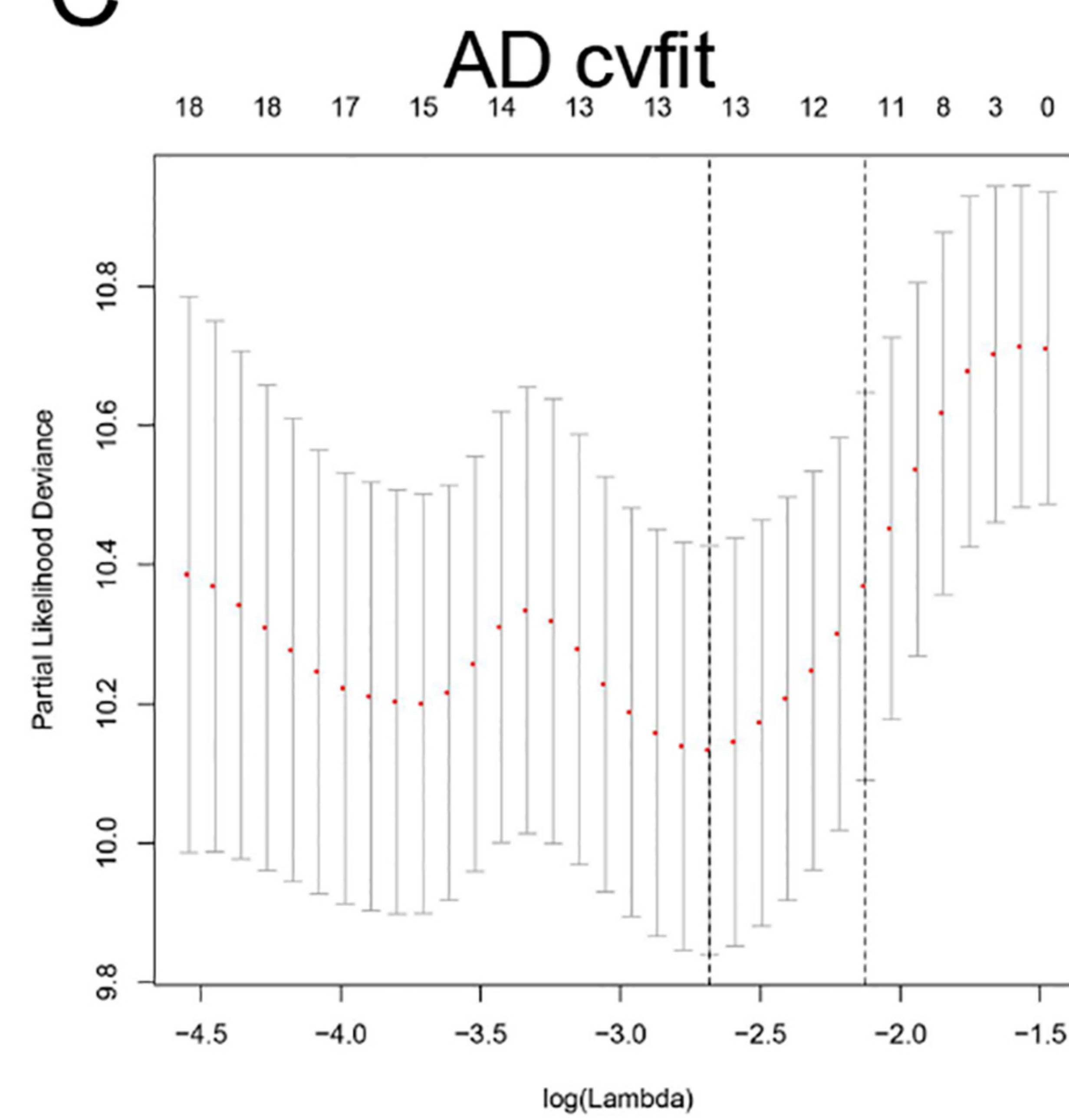**D**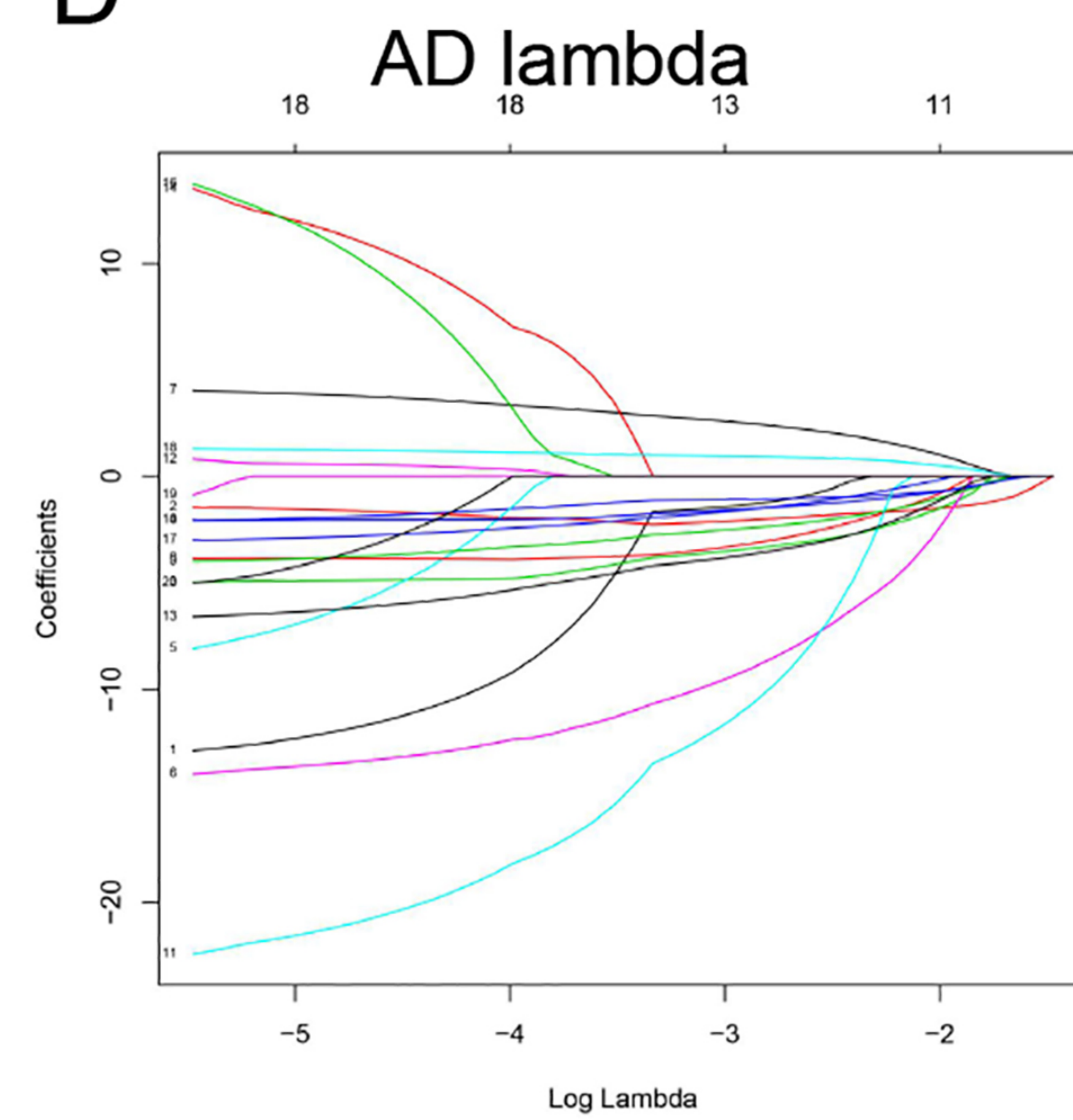**E**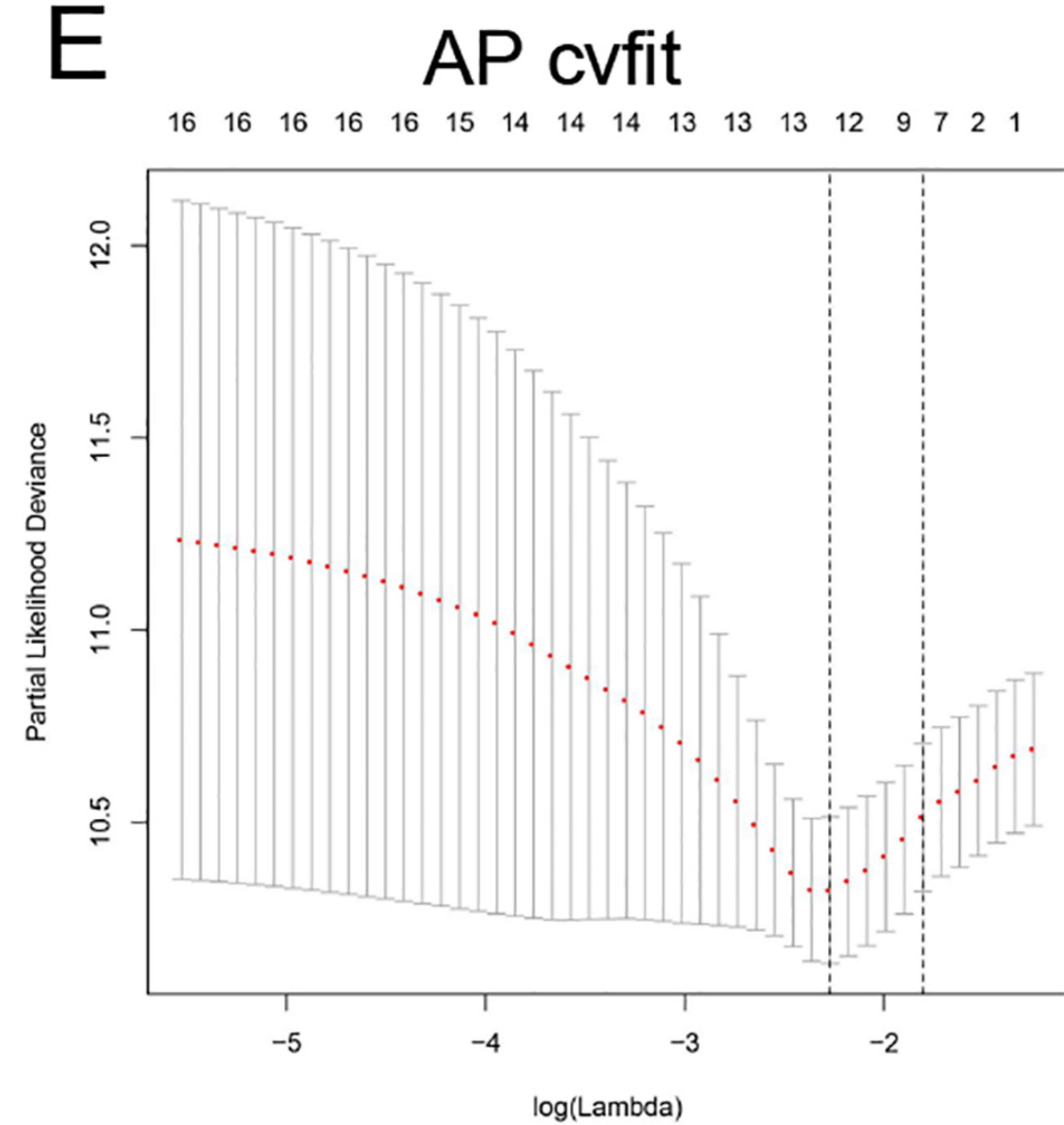**F**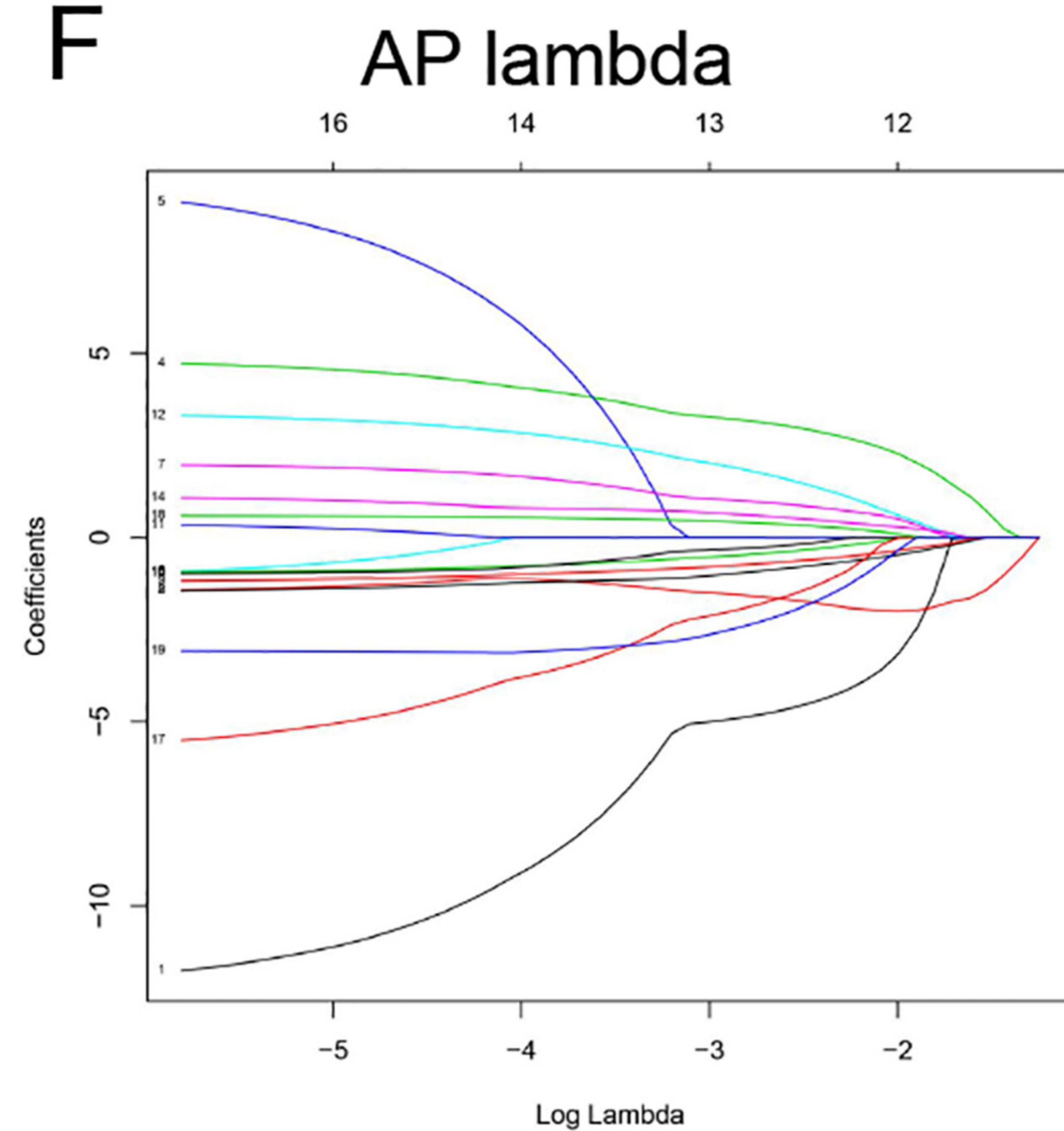**G**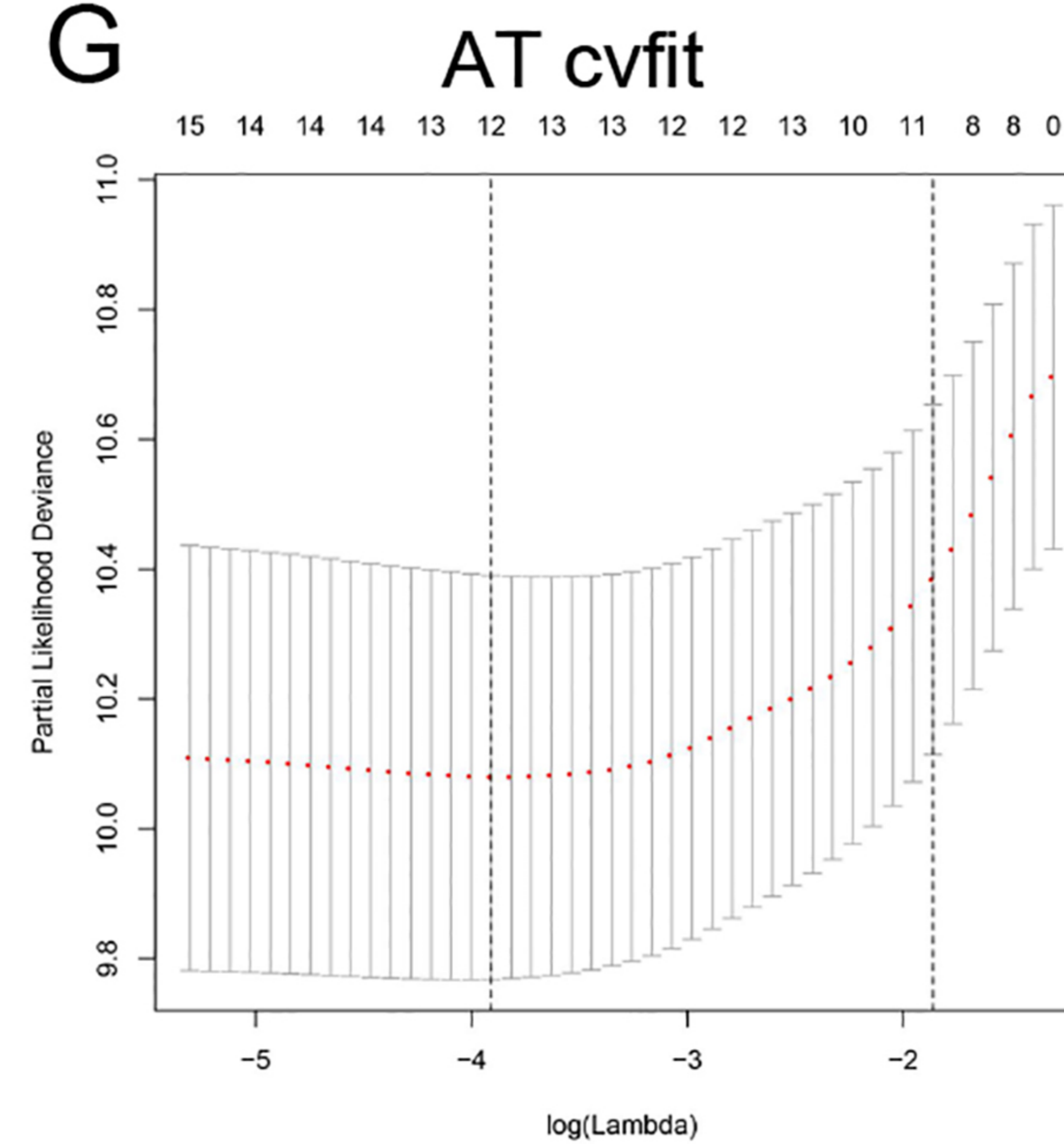**H**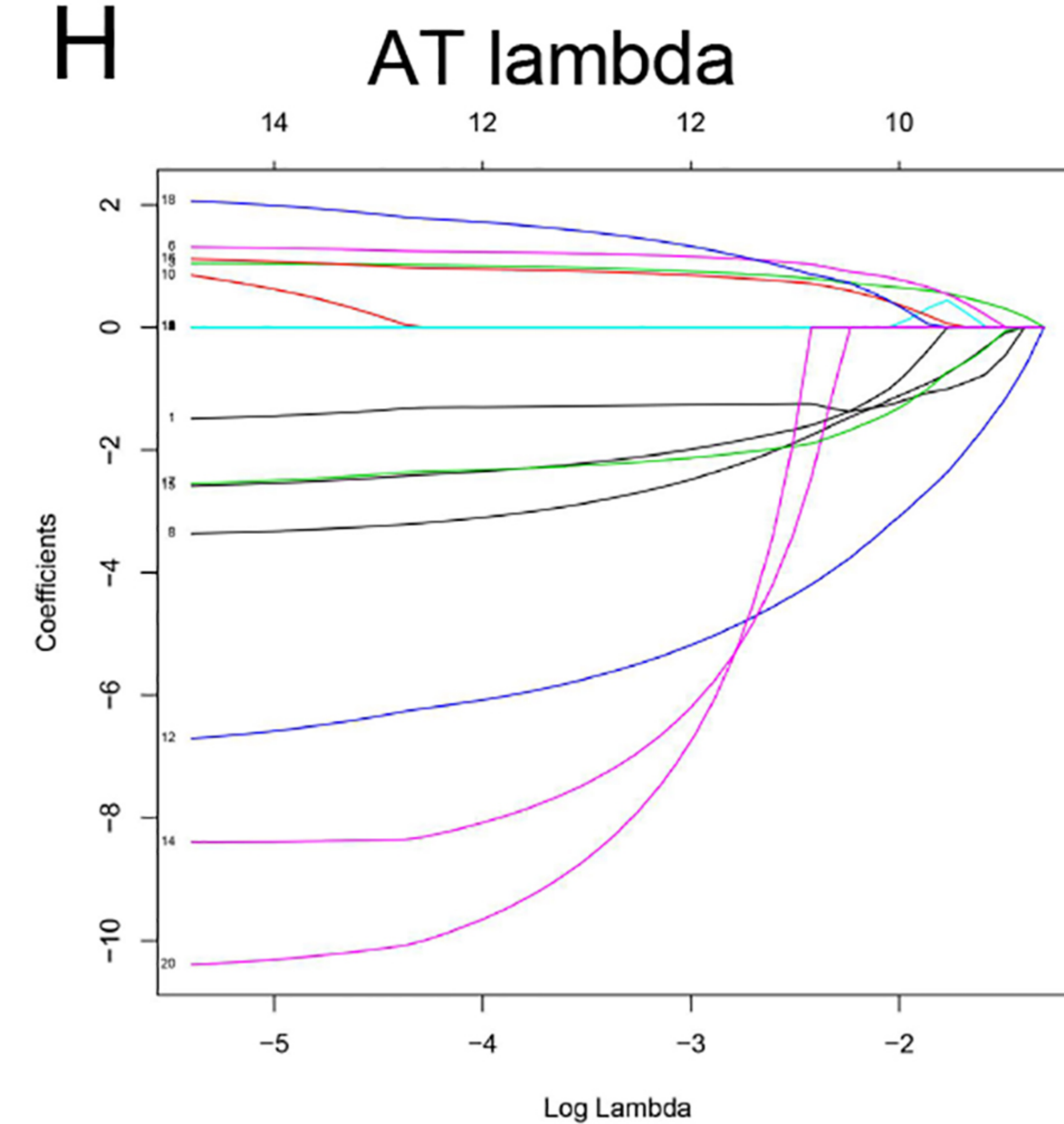**I**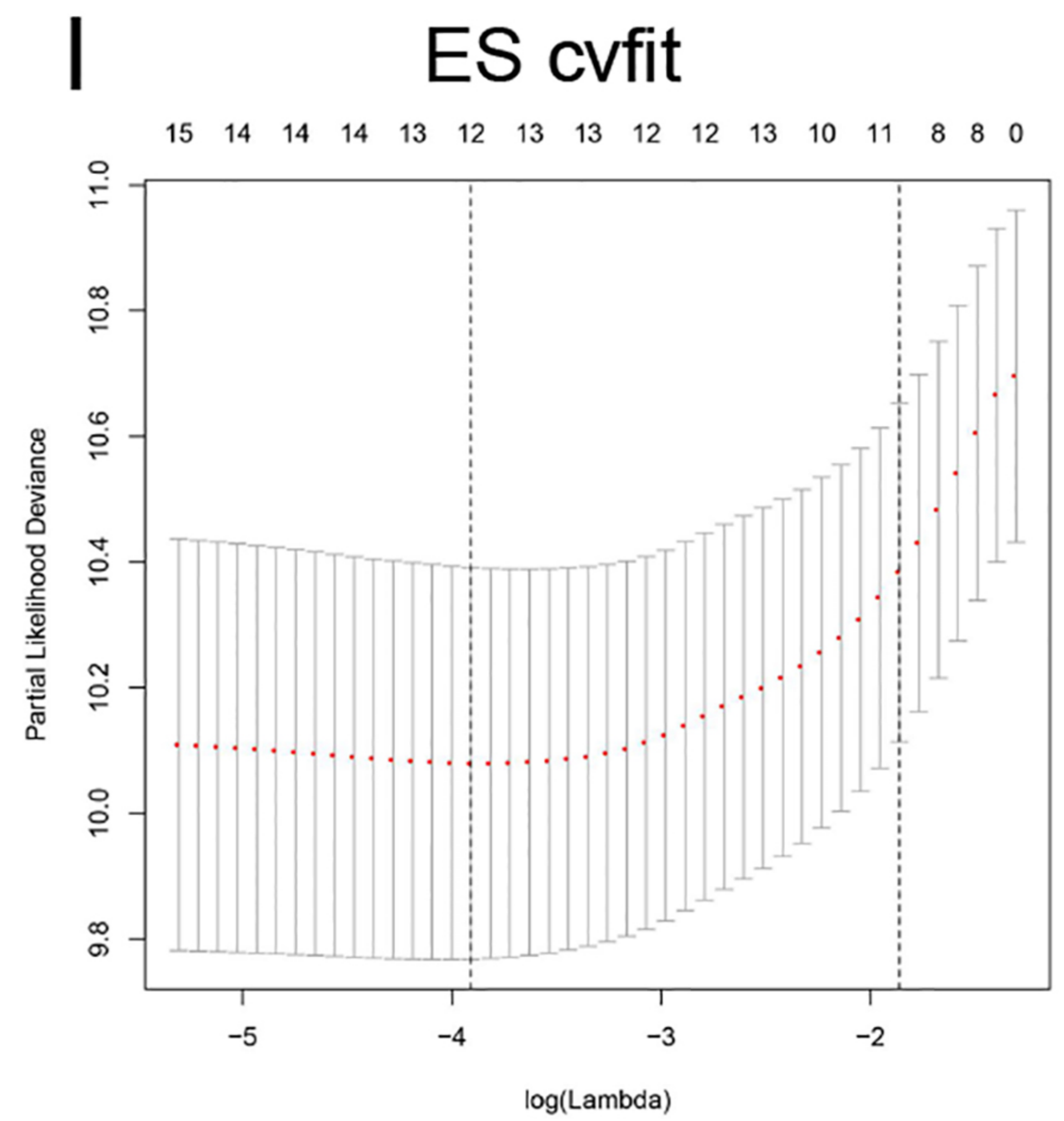**J**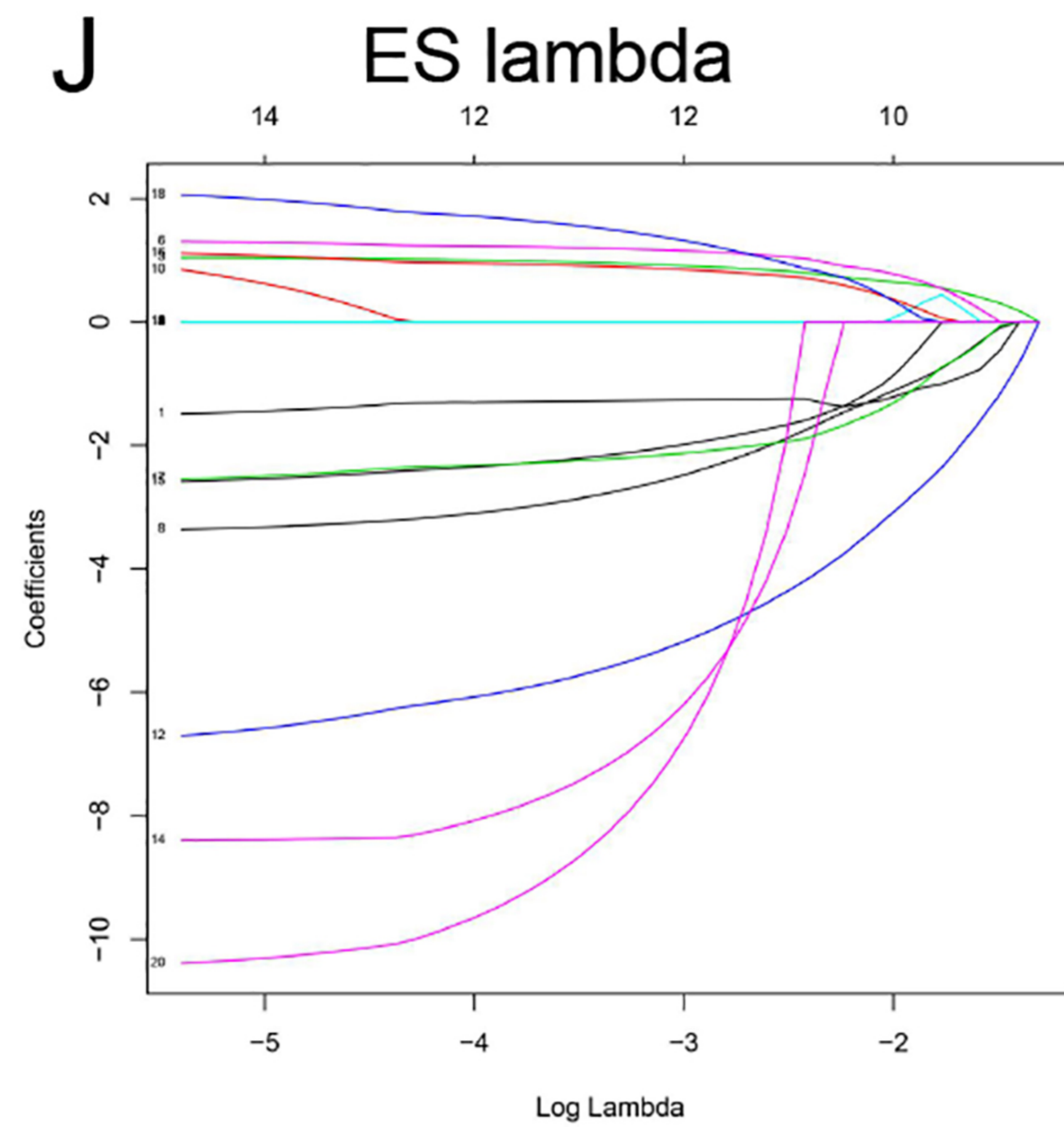**K**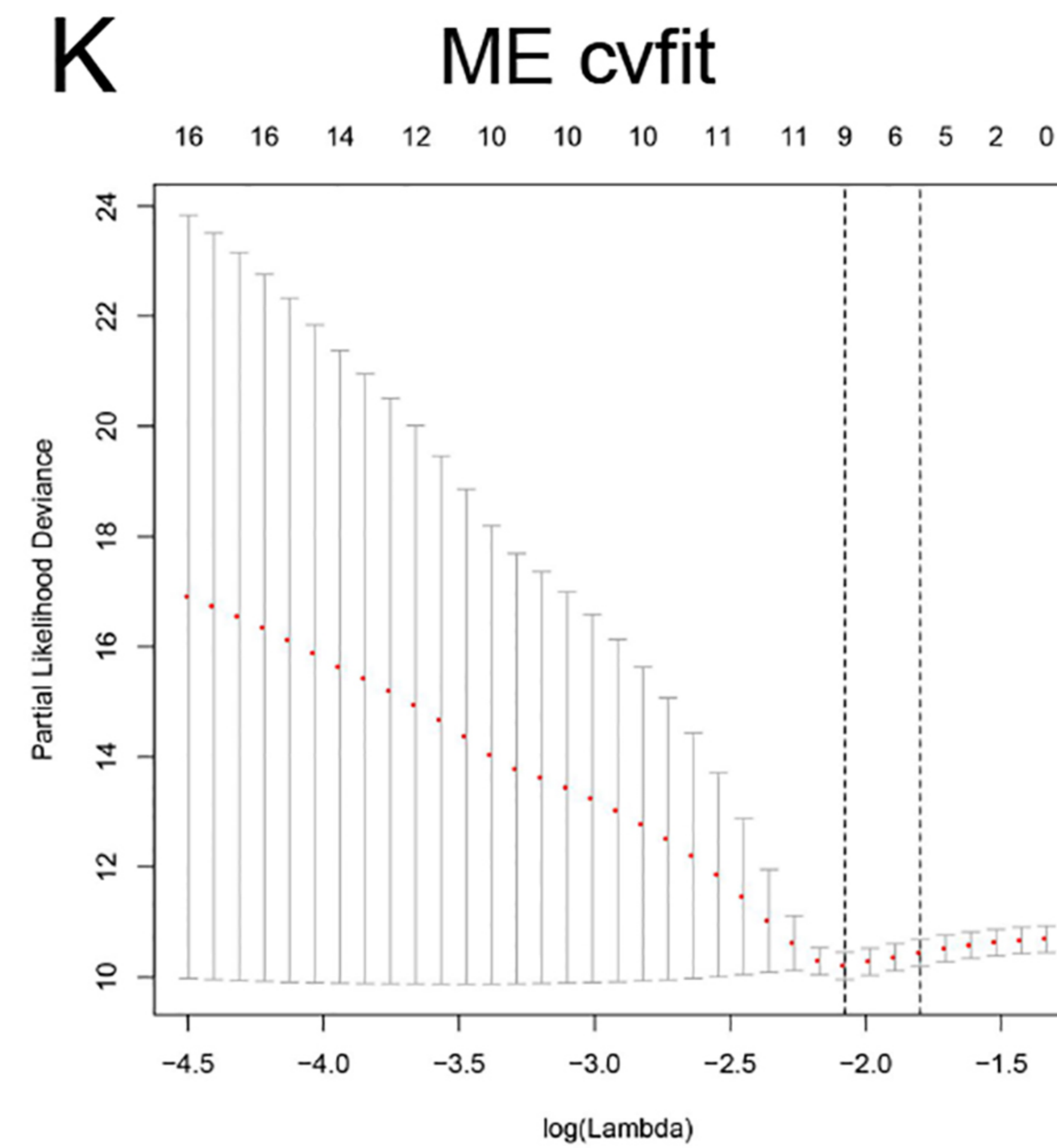**L**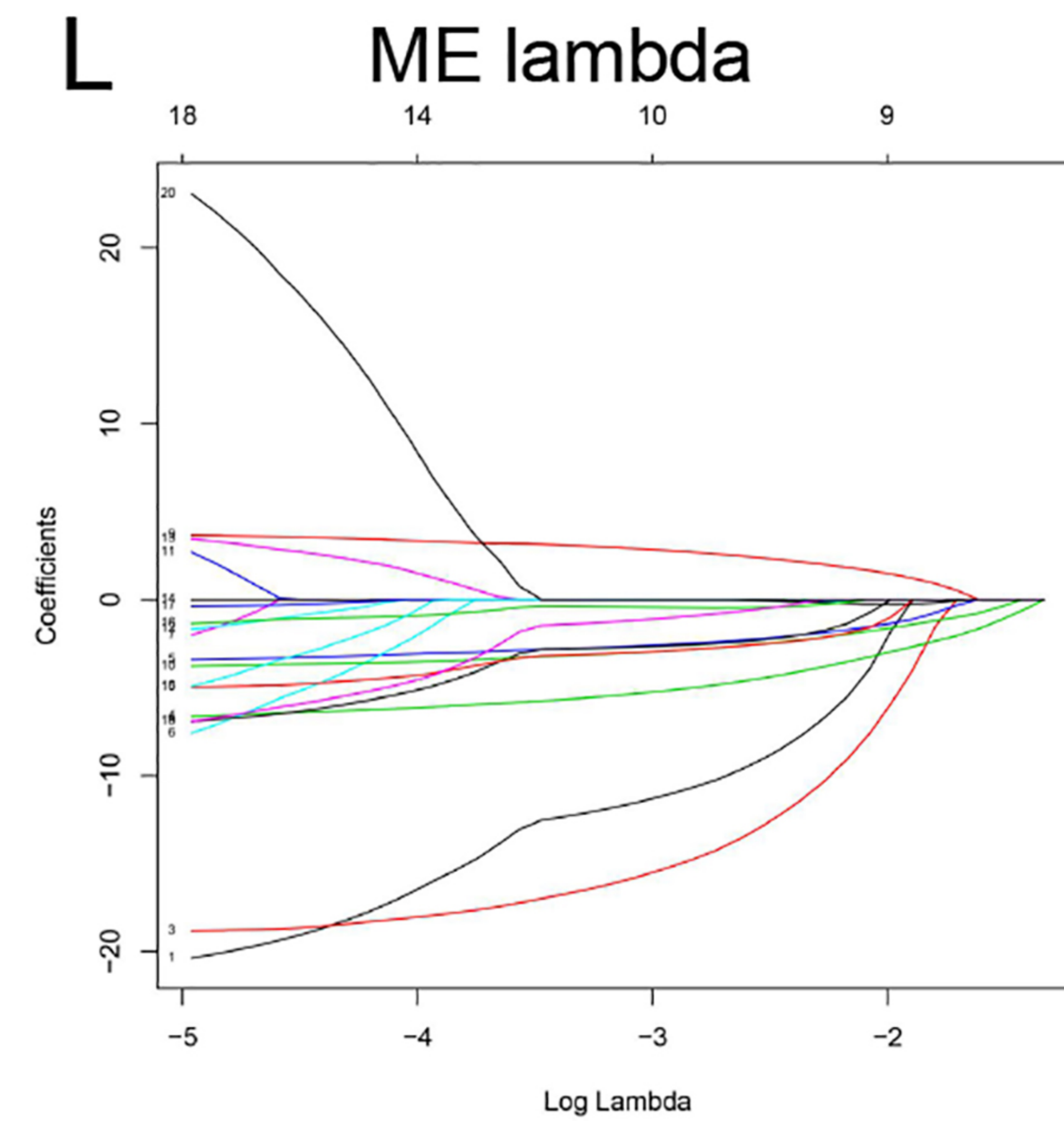**M**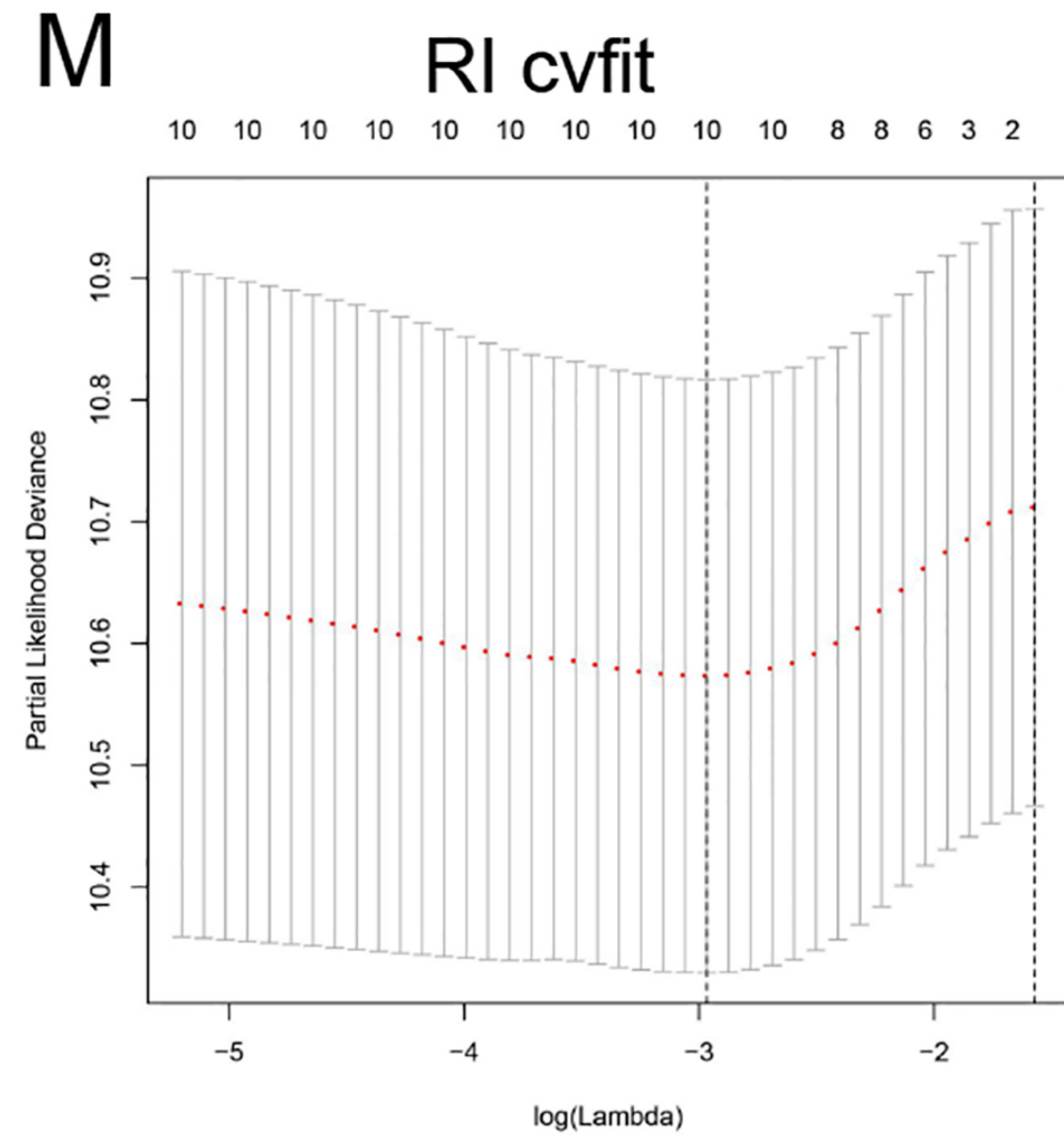**N**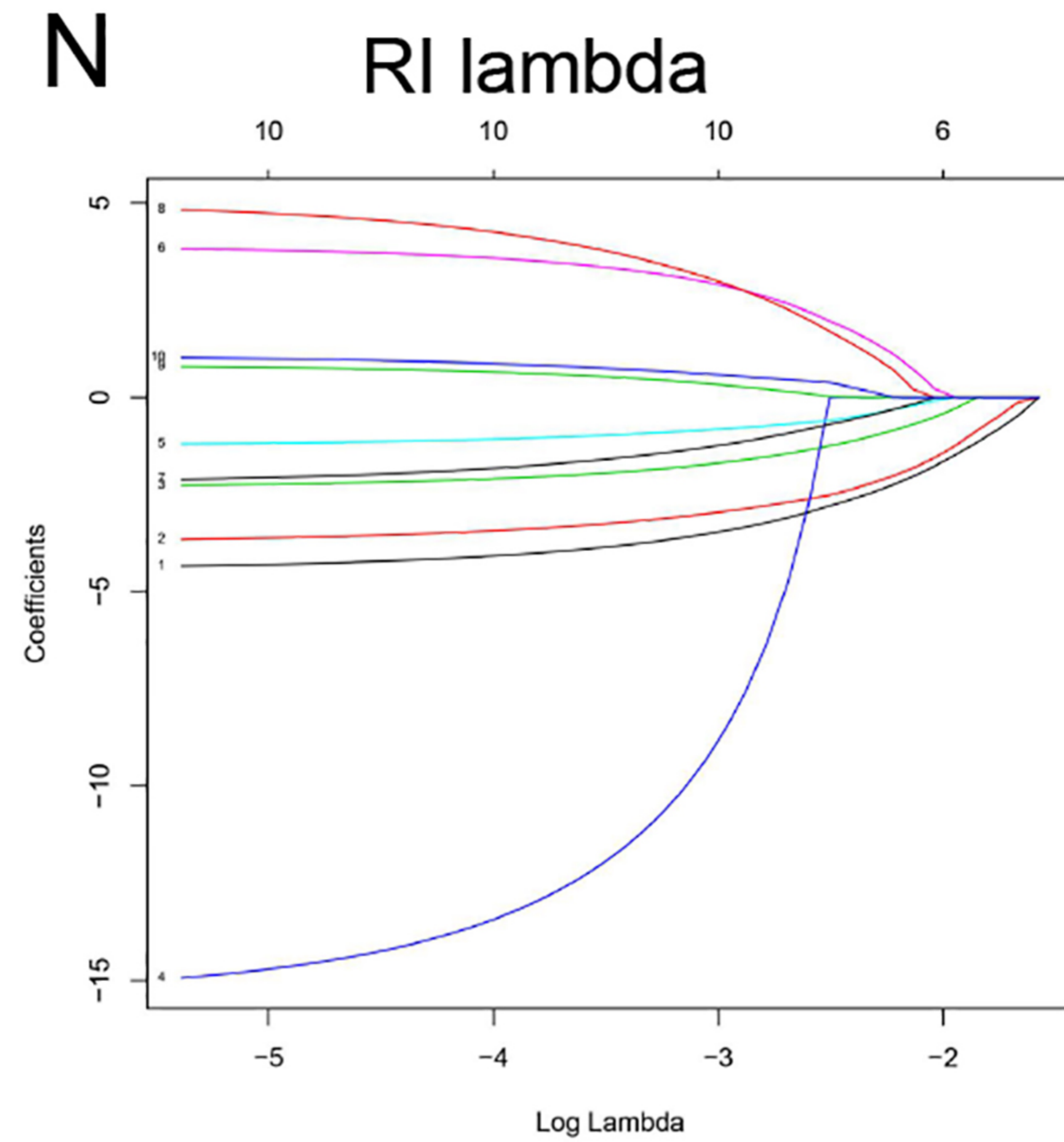**O**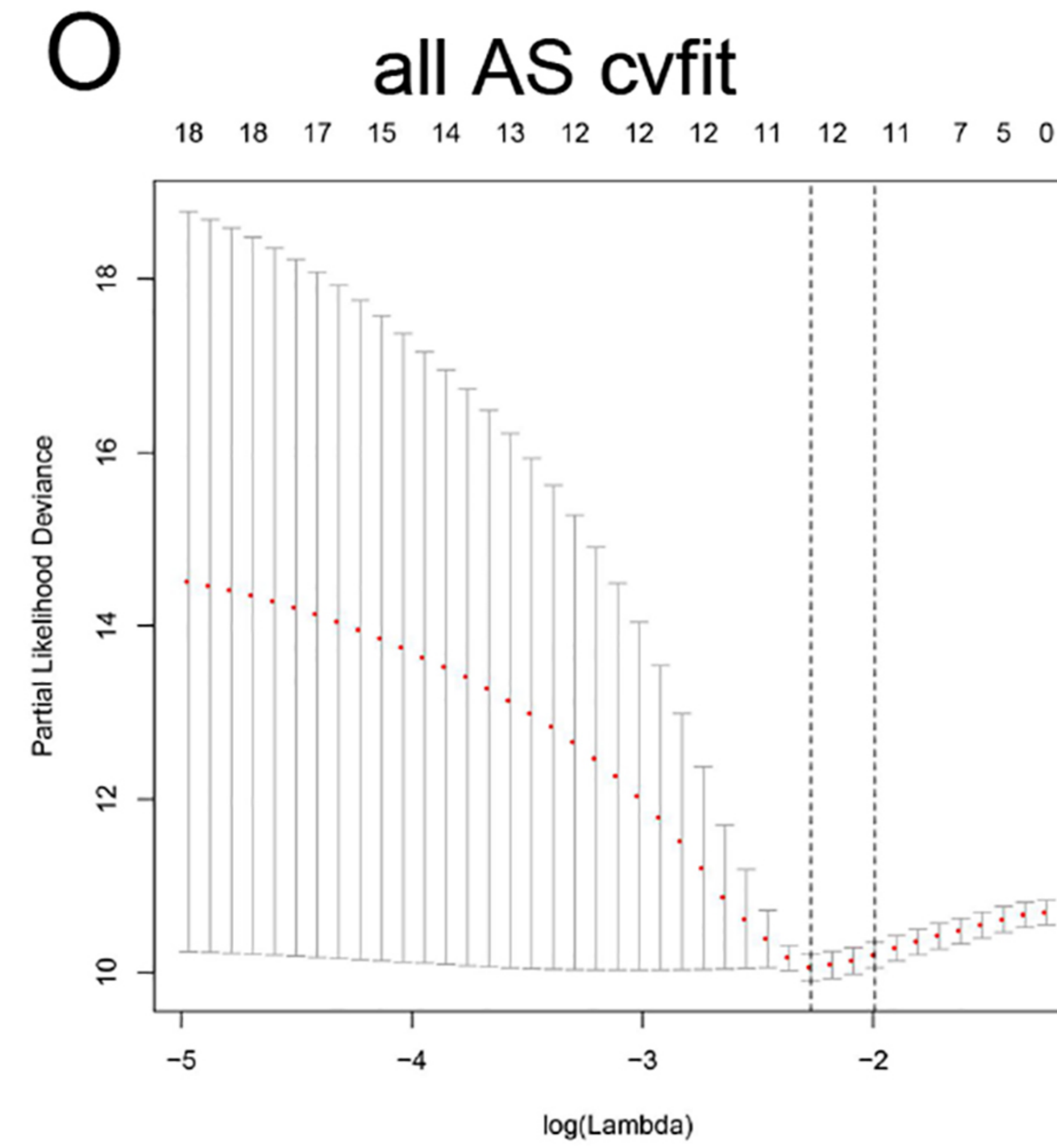**P**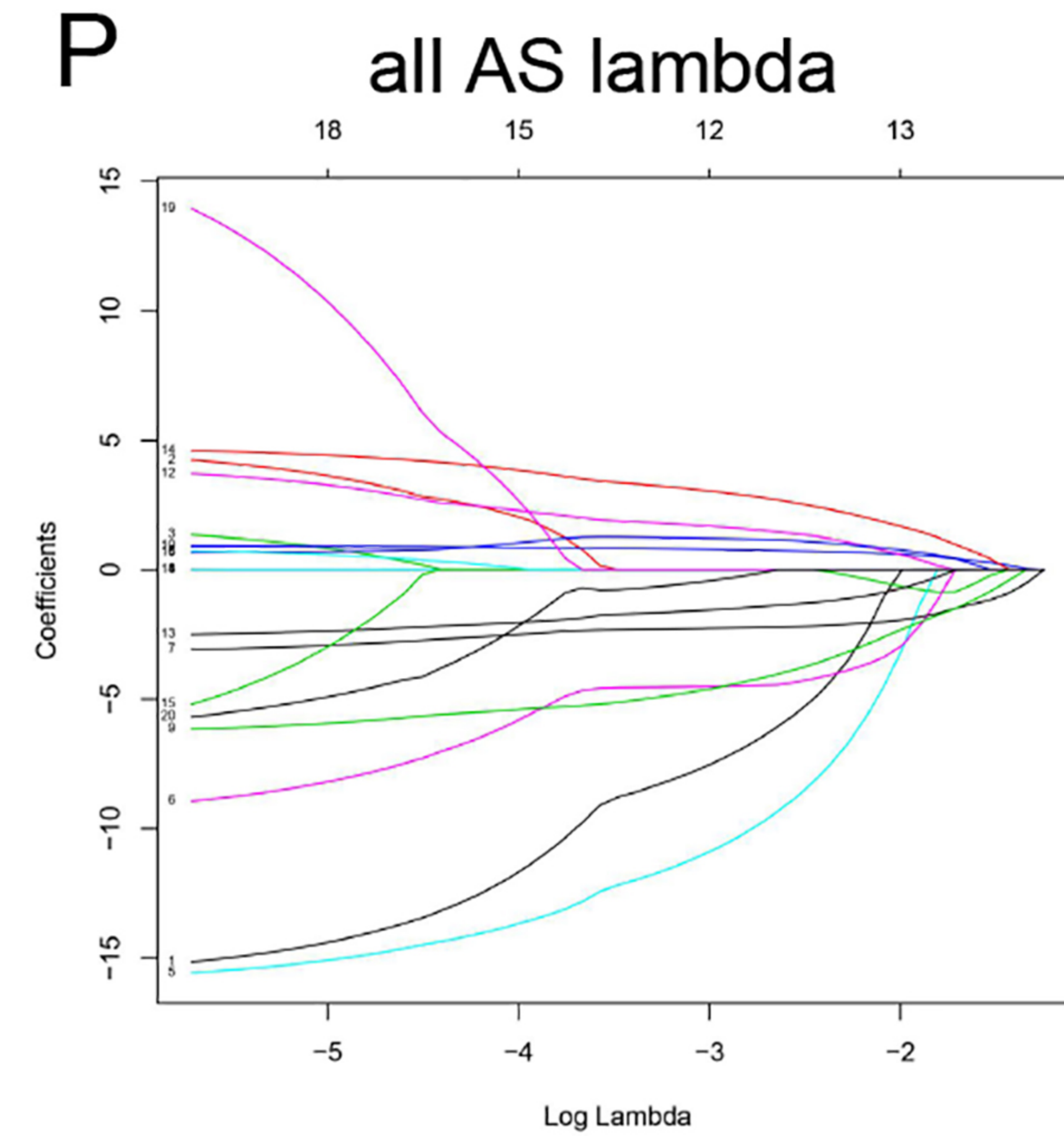

Supplement: Supplementary file 9 — Additional file 9: Figure S3. Selection of the optimal survival-related AS events used for construction of the final prediction model by LASSO regression. (A, C, E, G, I, K, M, O) Dotted vertical lines were drawn at the optimal values by using the minimum criteria. (B, D, F, H, J, L, N, P) LASSO coefficient profiles of the candidate survival-related AS events. [file 13578_2020_481_MOESM9_ESM.pdf]
