# Supplementary material for: Clusterization in acute myeloid leukemia based on prognostic alternative splicing signature to reveal the clinical characteristics in the bone marrow microenvironment
Source: Cell Biosci. 2020 Oct 12;10:118. doi: 10.1186/s13578-020-00481-5 (PMC7552347; doi:10.1186/s13578-020-00481-5)
Supplement: Supplementary file 4 — Additional file 4: Figure S2. Kaplan–Meier plots and ROC curves of prognostic predictors for AML patients. a-h Kaplan–Meier plots of prediction models associated with OS constructed with AA, AD, AP, AT, ES, ME, RI events and all types of AS events, respectively. i The ROC curves with AUCs of the predictive models for each AS markers. [file 13578_2020_481_MOESM4_ESM.pdf]

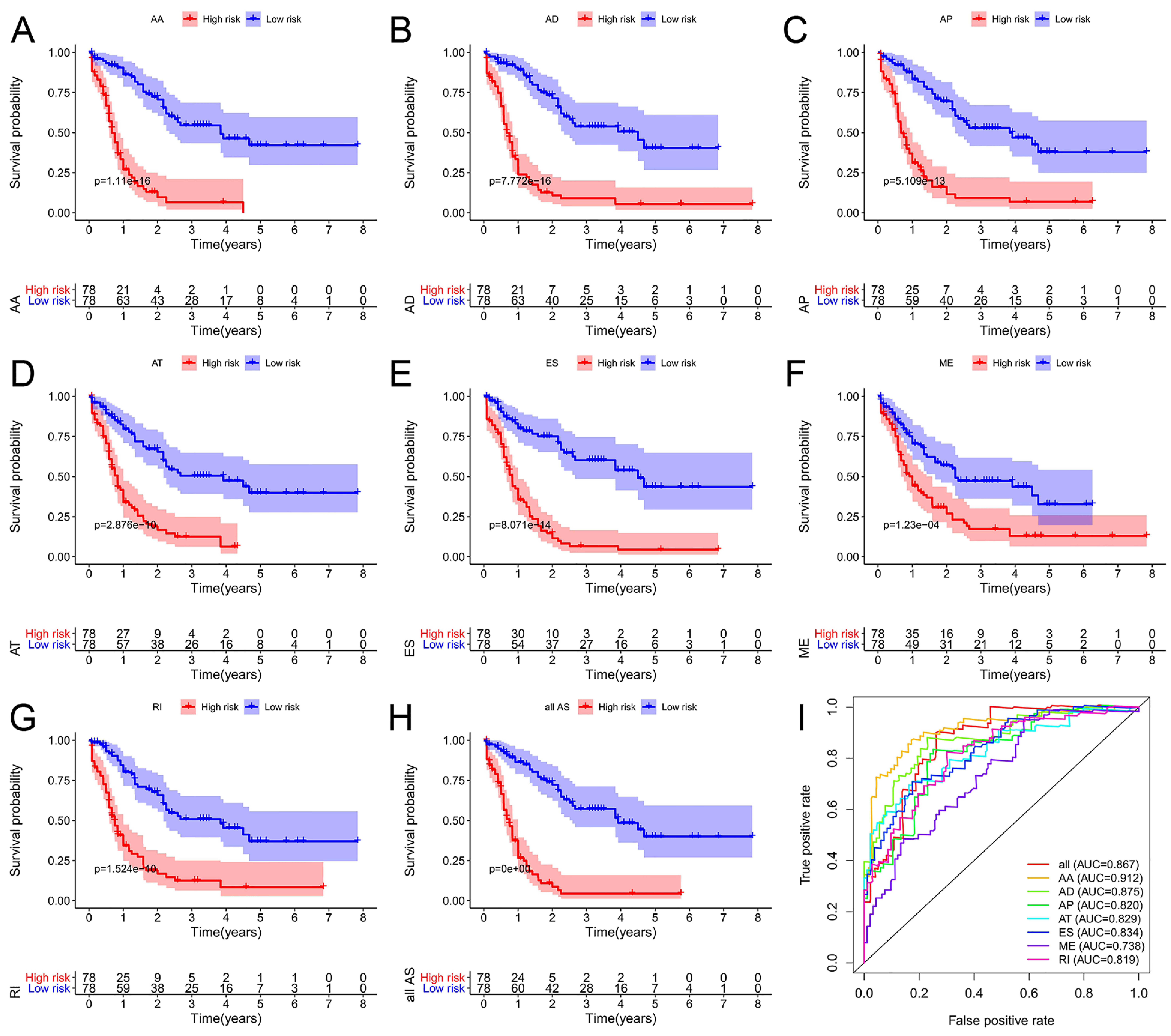
A
AA High risk Low risk
p=1.11e−16
High risk 78 21 4 2 1 0 0 0 0
Low risk 78 63 43 28 17 8 4 1 0
B
AD High risk Low risk
p=7.772e−16
High risk 78 21 7 5 3 2 1 1 0
Low risk 78 63 40 25 15 6 3 0 0
C
AP High risk Low risk
p=5.109e−13
High risk 78 25 7 4 3 2 1 0 0
Low risk 78 59 40 26 15 6 3 1 0
D
AT High risk Low risk
p=2.876e−10
High risk 78 27 9 4 2 0 0 0 0
Low risk 78 57 38 26 16 8 4 1 0
E
ES High risk Low risk
p=8.071e−14
High risk 78 30 10 3 2 2 1 0 0
Low risk 78 54 37 27 16 6 3 1 0
F
ME High risk Low risk
p=1.23e−04
High risk 78 35 16 9 6 3 2 1 0
Low risk 78 49 31 21 12 5 2 0 0
G
RI High risk Low risk
p=1.524e−10
High risk 78 25 9 5 2 1 1 0 0
Low risk 78 59 38 25 16 7 3 1 0
H
all AS High risk Low risk
p=0e+00
High risk 78 24 5 2 2 1 0 0 0
Low risk 78 60 42 28 16 7 4 1 0
Survival probability
Time(years)
I
True positive rate
False positive rate
all (AUC=0.867)
AA (AUC=0.912)
AD (AUC=0.875)
AP (AUC=0.820)
AT (AUC=0.829)
ES (AUC=0.834)
ME (AUC=0.738)
RI (AUC=0.819)
