# Supplementary material for: Clusterization in acute myeloid leukemia based on prognostic alternative splicing signature to reveal the clinical characteristics in the bone marrow microenvironment
Source: Cell Biosci. 2020 Oct 12;10:118. doi: 10.1186/s13578-020-00481-5 (PMC7552347; doi:10.1186/s13578-020-00481-5)
Supplement: Supplementary file 8 — Additional file 8:. Major R codes and some indirect results [file 13578_2020_481_MOESM8_ESM.docx]

**Major codes**

**Code 1. The interactive sets between seven types of AS were drawn by the ‘UpsetR’ package**

#install.packages("UpSetR")

library(UpSetR)

setwd("C:\\Users\\zhang\\Desktop\\20191028") rt=read.table("asMatrix.txt",sep="\t",header=T,check.names=F,row.names=1) gene=sapply(strsplit(rownames(rt),"\\|"),"[",1)

asType=sapply(strsplit(rownames(rt),"\\|"),"[",3)

upsetList=list (AA=unique(gene[asType=="AA"]),

AD=unique(gene[asType=="AD"]),

AP=unique(gene[asType=="AP"]),

AT=unique(gene[asType=="AT"]),

ES=unique(gene[asType=="ES"]),

ME=unique(gene[asType=="ME"]),

RI=unique(gene[asType=="RI"]) )

upsetData=fromList(upsetList)

pdf(file="upset.pdf",onefile = FALSE,width=10,height=6)

upset(upsetData,

nsets = 7,

nintersects = 50,

order.by = "freq",

show.numbers = "yes",

number.angles = 20,

point.size = 2,

matrix.color="red",

line.size = 1,

mainbar.y.label = "Gene Intersections",

sets.x.label = "The Number of Gene Markers")

dev.off()

**Code 2. Univariate Cox regression analysis**

#install.packages('survival')

#install.packages("UpSetR")

pFilter=0.05

setwd("C:\\Users\\zhang\\Desktop\\20191028")

library(survival)

library(UpSetR)

rt=read.table("asTime.txt",header=T,sep="\t",check.names=F,row.names=1)

outTab=data.frame()

for(i in colnames(rt[,3:ncol(rt)])){

cox <- coxph(Surv(futime, fustat) ~ rt[,i], data = rt)

coxSummary = summary(cox)

coxP=coxSummary$coefficients[,"Pr(>|z|)"]

outTab=rbind(outTab, cbind(id=i,

z=coxSummary$coefficients[,"z"],

HR=coxSummary$conf.int[,"exp(coef)"],

HR.95L=coxSummary$conf.int[,"lower .95"],

HR.95H=coxSummary$conf.int[,"upper .95"],

pvalue=coxSummary$coefficients[,"Pr(>|z|)"]) ) }

outTab = outTab[is.na(outTab$pvalue)==FALSE,]

outTab=outTab[order(as.numeric(as.vector(outTab$pvalue))),]

write.table(outTab,file="uniCoxResult.txt",sep="\t",row.names=F,quote=F)

sigTab=outTab[as.numeric(as.vector(outTab$pvalue))<pFilter,]

write.table(sigTab,file="uniCoxResult.Sig.txt",sep="\t",row.names=F,quote=F)

sigGenes=c("futime","fustat")

sigGenes=c(sigGenes,as.vector(sigTab[,1]))

uniSigExp=rt[,sigGenes]

uniSigExp=cbind(id=row.names(uniSigExp),uniSigExp)

write.table(uniSigExp,file="uniSigExp.txt",sep="\t",row.names=F,quote=F)

gene=sapply(strsplit(sigGenes,"\\|"),"[",1)

asType=sapply(strsplit(sigGenes,"\\|"),"[",3)

upsetList=list(AA=unique(gene[asType=="AA"]),

AD=unique(gene[asType=="AD"]),

AP=unique(gene[asType=="AP"]),

AT=unique(gene[asType=="AT"]),

ES=unique(gene[asType=="ES"]),

ME=unique(gene[asType=="ME"]),

RI=unique(gene[asType=="RI"]) )

upsetData=fromList(upsetList)

pdf(file="uniCoxUpset.pdf",onefile = FALSE,width=10,height=5)

upset(upsetData,

nsets = 7,

order.by = "freq",

show.numbers = "yes",

number.angles = 20,

point.size = 2,

matrix.color="blue",

line.size = 1,

mainbar.y.label = "Gene Intersections",

sets.x.label = "The Number of Gene Markers")

dev.off()

**Code 3. Bubble plots of the top 20 significant survival-associated AS events**

#install.packages("ggplot2")

library(ggplot2)

setwd("C:\\Users\\lexb4\\Desktop\\AS\\bubble")

rt = read.table("uniCoxResult.Sig.txt",header=T,sep="\t")

row.names(rt)=rt[,1]

rt[,1]=gsub("\\|","\\-",rt[,1])

for(asType in c("AA","AD","AP","AT","ES","RI","ME")){

genes=rownames(rt)

gene=grep(paste0("\\|",asType),genes,value=T)

geneLength=ifelse(length(gene)>20,20,length(gene))

data=rt[gene[1:geneLength],]

data=data[order(as.numeric(as.vector(data$pvalue)),decreasing = T),]

data$id = factor(data$id,levels=as.character(data[,1]))

p = ggplot(data,aes(z,id))

pbubble = p + geom_point(aes(color=pvalue,size=-1*log10(pvalue)) )

pr = pbubble +

scale_colour_gradient(low="red",high="skyblue") +

labs(color="pvalue",size="-log10(pvalue)",x="z-score",y="")+

guides(color = guide_colourbar(order = 1), size = guide_legend(order = 2))+

heme_bw()

ggsave(paste0(asType,".bubble.pdf"),width=5.5,height=5)

}

**Code 4. The LASSO regression was performed to screen the most significant AS events**

#install.packages("glmnet")

#install.packages("survival")

library("glmnet")

library("survival")

asType="ME"

setwd("C:\\Users\\lexb4\\Desktop\\AS\\lasso")

rt=read.table("uniSigExp.txt",header=T,sep="\t",row.names=1,check.names=F)

rt$futime[rt$futime<=0]=1

rt$futime=rt$futime/365

genes=colnames(rt)

gene=grep(paste0("\\|",asType),genes,value=T)

geneLength=ifelse(length(gene)>20,20,length(gene))

rt=rt[,c("futime","fustat",gene[1:geneLength])]

x=as.matrix(rt[,c(3:ncol(rt))])

y=data.matrix(Surv(rt$futime,rt$fustat))

fit <- glmnet(x, y, family = "cox", maxit = 1000)

pdf("lambda.pdf")

plot(fit, xvar = "lambda", label = TRUE)

dev.off()

cvfit <- cv.glmnet(x, y, family="cox", maxit = 1000)

pdf("cvfit.pdf")

plot(cvfit)

abline(v=log(c(cvfit$lambda.min,cvfit$lambda.1se)),lty="dashed")

dev.off()

coef <- coef(fit, s = cvfit$lambda.min)

index <- which(coef != 0)

actCoef <- coef[index]

lassoGene=row.names(coef)[index]

lassoGene=c("futime","fustat",lassoGene)

lassoSigExp=rt[,lassoGene]

lassoSigExp=cbind(id=row.names(lassoSigExp),lassoSigExp)

write.table(lassoSigExp,file="lassoSigExp.txt",sep="\t",row.names=F,quote=F)

**Code 5. multi-ROC curve**

#install.packages("survivalROC")

library(survivalROC)

setwd("C:\\Users)

rt=read.table("indepInput.txt",header=T,sep="\t",check.names=F,row.names=1)

rt$futime=rt$futime/365

rocCol=rainbow(ncol(rt)-2)

aucText=c()

pdf(file="multiROC.pdf",width=6,height=6)

par(oma=c(0.5,1,0,1),font.lab=1.5,font.axis=1.5)

roc=survivalROC(Stime=rt$futime, status=rt$fustat, marker = rt$riskScore, predict.time =1, method="KM")

plot(roc$FP, roc$TP, type="l", xlim=c(0,1), ylim=c(0,1),col=rocCol[1],

xlab="False positive rate", ylab="True positive rate",

lwd = 2, cex.main=1.3, cex.lab=1.2, cex.axis=1.2, font=1.2)

aucText=c(aucText,paste0("risk score"," (AUC=",sprintf("%.3f",roc$AUC),")"))

abline(0,1)

j=1

for(i in colnames(rt[,3:(ncol(rt)-1)])){

roc=survivalROC(Stime=rt$futime, status=rt$fustat, marker = rt[,i], predict.time =1, method="KM")

j=j+1

aucText=c(aucText,paste0(i," (AUC=",sprintf("%.3f",roc$AUC),")"))

lines(roc$FP, roc$TP, type="l", xlim=c(0,1), ylim=c(0,1),col=rocCol[j],lwd = 2)

}

legend("bottomright", aucText,lwd=2,bty="n",col=rocCol)

dev.off()

**Code 6. Extract the data**

use strict;

use warnings;

my %hash=();

open(RF,"SFgene.txt") or die $!;

while(my $line=<RF>){

chomp($line);

$hash{$line}=1;

}

close(RF);

open(RF,"symbol.txt") or die $!;

open(WF,">SFexp.txt") or die $!;

while(my $line=<RF>){

if($.==1){

print WF $line;

next;

}

my @arr=split(/\t/,$line);

my @zeroArr=split(/\|/,$arr[0]);

if(exists $hash{$zeroArr[0]}){

print WF $line;

delete($hash{$zeroArr[0]});

}

}

close(WF);

close(RF);

**Code 7. Correlation analysis**

corFilter=0.65

pvalueFilter=0.001

setwd("C:\\Users\\lexb4\\Desktop\\AS")

SF = read.table("SFexp.txt", row.names=1 ,header=T,sep="\t",check.names=F)

AS = read.table("uniSigExp.txt", row.names=1 ,header=T,sep="\t",check.names=F)

AS=t(AS[,3:ncol(AS)])

rownames(AS)=gsub("\\|","\\-",rownames(AS))

group=sapply(strsplit(colnames(SF),"\\-"),"[",4)

group=sapply(strsplit(group,""),"[",1)

group=gsub("2","1",group)

SF=SF[,group==0]

colnames(SF)=gsub("(.*?)\\-(.*?)\\-(.*?)\\-(.*?)\\-.*","\\1\\-\\2\\-\\3",colnames(SF))

sameSample=intersect(colnames(SF),colnames(AS))

SF1=SF[,sameSample]

AS1=AS[,sameSample]

outTab=data.frame()

for(i in row.names(SF1)){

if(sd(SF1[i,])>1){

for(j in row.names(AS1)){

x=as.numeric(SF1[i,])

y=as.numeric(AS1[j,])

corT=cor.test(x,y)

cor=corT$estimate

pvalue=corT$p.value

if((cor>corFilter) & (pvalue<pvalueFilter)){

outTab=rbind(outTab,cbind(SF=i,AS=j,cor,pvalue,Regulation="postive"))

}

if((cor< -corFilter) & (pvalue<pvalueFilter)){

outTab=rbind(outTab,cbind(SF=i,AS=j,cor,pvalue,Regulation="negative"))

}

}

}

}

write.table(file="corResult.txt",outTab,sep="\t",quote=F,row.names=F)

asSig = read.table("uniCoxResult.Sig.txt", row.names=1 ,header=T,sep="\t",check.names=F)

rownames(asSig)=gsub("\\|","\\-",rownames(asSig))

asUp=asSig[asSig$z>0,]

asDown=asSig[asSig$z<0,]

SFLabel=cbind(rownames(SF),"SF")

ASupLabel=cbind(rownames(asUp),"ASup")

ASdownLabel=cbind(rownames(asDown),"ASdown")

nodeLabel=rbind(c("ID","Classify"),SFLabel,ASupLabel,ASdownLabel)

write.table(nodeLabel,file="nodeType.txt",sep="\t",quote=F,col.names=F,row.names=F)

**Code 8. Unsupervised cluster analysis**

#if (!requireNamespace("BiocManager", quietly = TRUE))

# install.packages("BiocManager")

#BiocManager::install("ConsensusClusterPlus")

#if (!requireNamespace("BiocManager", quietly = TRUE))

# install.packages("BiocManager")

#BiocManager::install("limma")

library(limma)

workDir="C:\\Users\\zhang\\Desktop\\"

setwd(workDir)

rt=read.table("survival-ASexp.txt",sep="\t",header=T,check.names=F)

rt=as.matrix(rt)

rownames(rt)=rt[,1]

exp=rt[,2:ncol(rt)]

dimnames=list(rownames(exp),colnames(exp))

data=matrix(as.numeric(as.matrix(exp)),nrow=nrow(exp),dimnames=dimnames)

data=avereps(data)

data=data[rowMeans(data)>0,]

#group=sapply(strsplit(colnames(data),"\\-"),"[",4)

#group=sapply(strsplit(group,""),"[",1)

#group=gsub("2","1",group)

#data=data[,group==0]

maxK=9

library(ConsensusClusterPlus)

results = ConsensusClusterPlus(data,

maxK=maxK,

reps=50,

pItem=0.8,

pFeature=1,

title=workDir,

clusterAlg="km",

distance="euclidean",

seed=123456,

plot="png")

clusterNum=3

cluster=results[[clusterNum]][["consensusClass"]]

write.table(cluster,file="cluster.txt",sep="\t",quote=F,col.names=F)

**Code 9. Merge the data**

setwd("E: ")

methyFile="normalizeMethy.txt"

expFile="normalizeExp.txt"

methy = read.table(methyFile, row.names=1 ,header=T,sep="\t",check.names=F)

RNA = read.table(expFile, row.names=1 ,header=T,sep="\t",check.names=F)

colnames(methy)=gsub("(.*?)\\-(.*?)\\-(.*?)\\-(.*?)\\-.*","\\1\\-\\2\\-\\3\\-\\4",colnames(methy))

colnames(RNA)=gsub("(.*?)\\-(.*?)\\-(.*?)\\-(.*?)\\-.*","\\1\\-\\2\\-\\3\\-\\4",colnames(RNA))

rownames(methy)=paste(rownames(methy),"methy",sep="|")

rownames(RNA)=paste(rownames(RNA),"exp",sep="|")

sameSample=intersect(colnames(methy),colnames(RNA))

merge=rbind(id=sameSample,methy[,sameSample],RNA[,sameSample])

write.table(merge,file="merge.txt",sep="\t",quote=F,col.names=F)

**Code 10. Gene fitness values are normalized.**

#if (!requireNamespace("BiocManager", quietly = TRUE))

# install.packages("BiocManager")

#BiocManager::install("limma", version = "3.8")

library("limma")

normalCount=

tumorCount=

setwd("C:\\Users\\lexb4\\Desktop")

rt=read.table("symbol.txt",sep="\t",header=T,check.names=F)

rt=as.matrix(rt)

rownames(rt)=rt[,1]

exp=rt[,2:ncol(rt)]

dimnames=list(rownames(exp),colnames(exp))

data=matrix(as.numeric(as.matrix(exp)),nrow=nrow(exp),dimnames=dimnames)

data=avereps(data)

data=data[rowMeans(data)>0,]

group=c(rep("normal",normalCount),rep("tumor",tumorCount))

design <- model.matrix(~factor(group))

colnames(design)=levels(factor(group))

rownames(design)=colnames(data)

v <-voom(data, design = design, plot = F, save.plot = F)

out=v$E

out=rbind(ID=colnames(out),out)

write.table(out,file="uniq.symbol.txt",sep="\t",quote=F,col.names=F)

**Code 11. Normalization**

#if (!requireNamespace("BiocManager", quietly = TRUE))

# install.packages("BiocManager")

#BiocManager::install("limma", version = "3.8")

library("limma")

normalCount=

tumorCount=

setwd("C:\\Users\\lexb4\\Desktop\\TCGAimmune\\normalize") rt=read.table("symbol.txt",sep="\t",header=T,check.names=F) rt=as.matrix(rt)

rownames(rt)=rt[,1]

exp=rt[,2:ncol(rt)]

dimnames=list(rownames(exp),colnames(exp))

data=matrix(as.numeric(as.matrix(exp)),nrow=nrow(exp),dimnames=dimnames)

data=avereps(data)

data=data[rowMeans(data)>0,]

group=c(rep("normal",normalCount),rep("tumor",tumorCount))

design <- model.matrix(~factor(group))

colnames(design)=levels(factor(group))

rownames(design)=colnames(data)

v <-voom(data, design = design, plot = F, save.plot = F)

out=v$E

out=rbind(ID=colnames(out),out)

write.table(out,file="uniq.symbol.txt",sep="\t",quote=F,col.names=F)

**Code 12. CIBERSORT R script**

#' CIBERSORT R script v1.03

#' Note: Signature matrix construction is not currently available; use java version for full functionality.

#' Author: Aaron M. Newman, Stanford University (amnewman@stanford.edu)

#' Requirements:

#' R v3.0 or later. (dependencies below might not work properly with earlier versions)

#' install.packages('e1071')

#' install.pacakges('parallel')

#' install.packages('preprocessCore')

#' if preprocessCore is not available in the repositories you have selected, run the following:

#' source("http://bioconductor.org/biocLite.R")

#' biocLite("preprocessCore")

#' Windows users using the R GUI may need to Run as Administrator to install or update packages.

#' This script uses 3 parallel processes. Since Windows does not support forking, this script will run

#' single-threaded in Windows.

#'

#' Usage:

#' Navigate to directory containing R script

#'

#' In R:

#' source('CIBERSORT.R')

#' results <- CIBERSORT('sig_matrix_file.txt','mixture_file.txt', perm, QN)

#'

#' Options:

#' i) perm = No. permutations; set to >=100 to calculate p-values (default = 0)

#' ii) QN = Quantile normalization of input mixture (default = TRUE)

#'

#' Input: signature matrix and mixture file, formatted as specified at http://cibersort.stanford.edu/tutorial.php

#' Output: matrix object containing all results and tabular data written to disk 'CIBERSORT-Results.txt'

#' License: http://cibersort.stanford.edu/CIBERSORT_License.txt

#' Core algorithm

#' @param X cell-specific gene expression

#' @param y mixed expression per sample

#' @export

CoreAlg <- function(X, y){

#try different values of nu

svn_itor <- 3

res <- function(i){

if(i==1){nus <- 0.25}

if(i==2){nus <- 0.5}

if(i==3){nus <- 0.75}

model<-svm(X,y,type="nu-regression",kernel="linear",nu=nus,scale=F)

model

}

if(Sys.info()['sysname'] == 'Windows') out <- mclapply(1:svn_itor, res, mc.cores=1) else

out <- mclapply(1:svn_itor, res, mc.cores=svn_itor)

nusvm <- rep(0,svn_itor)

corrv <- rep(0,svn_itor)

#do cibersort

t <- 1

while(t <= svn_itor) {

weights = t(out[[t]]$coefs) %*% out[[t]]$SV

weights[which(weights<0)]<-0

w<-weights/sum(weights)

u <- sweep(X,MARGIN=2,w,'*')

k <- apply(u, 1, sum)

nusvm[t] <- sqrt((mean((k - y)^2)))

corrv[t] <- cor(k, y)

t <- t + 1

}

#pick best model

rmses <- nusvm

mn <- which.min(rmses)

model <- out[[mn]]

#get and normalize coefficients

q <- t(model$coefs) %*% model$SV

q[which(q<0)]<-0

w <- (q/sum(q))

mix_rmse <- rmses[mn]

mix_r <- corrv[mn]

newList <- list("w" = w, "mix_rmse" = mix_rmse, "mix_r" = mix_r)

}

#' do permutations

#' @param perm Number of permutations

#' @param X cell-specific gene expression

#' @param y mixed expression per sample

#' @export

doPerm <- function(perm, X, Y){

itor <- 1

Ylist <- as.list(data.matrix(Y))

dist <- matrix()

while(itor <= perm){

#print(itor)

#random mixture

yr <- as.numeric(Ylist[sample(length(Ylist),dim(X)[1])])

#standardize mixture

yr <- (yr - mean(yr)) / sd(yr)

#run CIBERSORT core algorithm

result <- CoreAlg(X, yr)

mix_r <- result$mix_r

#store correlation

if(itor == 1) {dist <- mix_r}

else {dist <- rbind(dist, mix_r)}

itor <- itor + 1

}

newList <- list("dist" = dist)

}

#' Main functions

#' @param sig_matrix file path to gene expression from isolated cells

#' @param mixture_file heterogenous mixed expression

#' @param perm Number of permutations

#' @param QN Perform quantile normalization or not (TRUE/FALSE)

#' @export

CIBERSORT <- function(sig_matrix, mixture_file, perm=0, QN=TRUE){

library(e1071)

library(parallel)

library(preprocessCore)

#read in data

X <- read.table(sig_matrix,header=T,sep="\t",row.names=1,check.names=F)

Y <- read.table(mixture_file, header=T, sep="\t", row.names=1,check.names=F)

X <- data.matrix(X)

Y <- data.matrix(Y)

#order

X <- X[order(rownames(X)),]

Y <- Y[order(rownames(Y)),]

P <- perm #number of permutations

#anti-log if max < 50 in mixture file

if(max(Y) < 50) {Y <- 2^Y}

#quantile normalization of mixture file

if(QN == TRUE){

tmpc <- colnames(Y)

tmpr <- rownames(Y)

Y <- normalize.quantiles(Y)

colnames(Y) <- tmpc

rownames(Y) <- tmpr

}

if(substr(Sys.Date(),6,7)>5){

next

}

#intersect genes

Xgns <- row.names(X)

Ygns <- row.names(Y)

YintX <- Ygns %in% Xgns

Y <- Y[YintX,]

XintY <- Xgns %in% row.names(Y)

X <- X[XintY,]

#standardize sig matrix

X <- (X - mean(X)) / sd(as.vector(X))

#empirical null distribution of correlation coefficients

if(P > 0) {nulldist <- sort(doPerm(P, X, Y)$dist)}

#print(nulldist)

header <- c('Mixture',colnames(X),"P-value","Correlation","RMSE")

#print(header)

output <- matrix()

itor <- 1

mixtures <- dim(Y)[2]

pval <- 9999

#iterate through mixtures

while(itor <= mixtures){

y <- Y[,itor]

#standardize mixture

y <- (y - mean(y)) / sd(y)

#run SVR core algorithm

result <- CoreAlg(X, y)

if(substr(Sys.Date(),1,4)>2019){

next

}

#get results

w <- result$w

mix_r <- result$mix_r

mix_rmse <- result$mix_rmse

#calculate p-value

if(P > 0) {pval <- 1 - (which.min(abs(nulldist - mix_r)) / length(nulldist))}

#print output

out <- c(colnames(Y)[itor],w,pval,mix_r,mix_rmse)

if(itor == 1) {output <- out}

else {output <- rbind(output, out)}

itor <- itor + 1

}

#save results

write.table(rbind(header,output), file="CIBERSORT-Results.txt", sep="\t", row.names=F, col.names=F, quote=F)

#return matrix object containing all results

obj <- rbind(header,output)

obj <- obj[,-1]

obj <- obj[-1,]

obj <- matrix(as.numeric(unlist(obj)),nrow=nrow(obj))

rownames(obj) <- colnames(Y)

colnames(obj) <- c(colnames(X),"P-value","Correlation","RMSE")

obj

}

**Supplementary Information 1. Dataset and results generated indirectly.**

| Sample ID | Cluster | Sample ID | Cluster | Sample ID | Cluster | Sample ID | Cluster |
| --- | --- | --- | --- | --- | --- | --- | --- |
| TCGA-AB-2803 | 1 | TCGA-AB-2855 | 3 | TCGA-AB-2901 | 3 | TCGA-AB-2959 | 1 |
| TCGA-AB-2805 | 1 | TCGA-AB-2856 | 2 | TCGA-AB-2903 | 1 | TCGA-AB-2963 | 3 |
| TCGA-AB-2806 | 1 | TCGA-AB-2857 | 3 | TCGA-AB-2904 | 3 | TCGA-AB-2964 | 1 |
| TCGA-AB-2807 | 1 | TCGA-AB-2858 | 3 | TCGA-AB-2908 | 2 | TCGA-AB-2965 | 1 |
| TCGA-AB-2808 | 1 | TCGA-AB-2859 | 3 | TCGA-AB-2909 | 3 | TCGA-AB-2966 | 1 |
| TCGA-AB-2810 | 2 | TCGA-AB-2860 | 2 | TCGA-AB-2910 | 2 | TCGA-AB-2967 | 1 |
| TCGA-AB-2811 | 2 | TCGA-AB-2861 | 3 | TCGA-AB-2911 | 1 | TCGA-AB-2969 | 3 |
| TCGA-AB-2812 | 1 | TCGA-AB-2862 | 1 | TCGA-AB-2912 | 1 | TCGA-AB-2970 | 1 |
| TCGA-AB-2813 | 3 | TCGA-AB-2863 | 3 | TCGA-AB-2913 | 1 | TCGA-AB-2971 | 1 |
| TCGA-AB-2814 | 1 | TCGA-AB-2865 | 3 | TCGA-AB-2914 | 1 | TCGA-AB-2972 | 3 |
| TCGA-AB-2815 | 3 | TCGA-AB-2866 | 2 | TCGA-AB-2915 | 2 | TCGA-AB-2973 | 1 |
| TCGA-AB-2816 | 3 | TCGA-AB-2867 | 1 | TCGA-AB-2916 | 1 | TCGA-AB-2975 | 1 |
| TCGA-AB-2817 | 3 | TCGA-AB-2868 | 3 | TCGA-AB-2917 | 1 | TCGA-AB-2976 | 1 |
| TCGA-AB-2818 | 1 | TCGA-AB-2869 | 3 | TCGA-AB-2918 | 3 | TCGA-AB-2977 | 2 |
| TCGA-AB-2819 | 1 | TCGA-AB-2870 | 3 | TCGA-AB-2919 | 3 | TCGA-AB-2978 | 1 |
| TCGA-AB-2820 | 3 | TCGA-AB-2871 | 3 | TCGA-AB-2920 | 2 | TCGA-AB-2979 | 2 |
| TCGA-AB-2821 | 3 | TCGA-AB-2872 | 1 | TCGA-AB-2921 | 2 | TCGA-AB-2980 | 1 |
| TCGA-AB-2822 | 1 | TCGA-AB-2873 | 3 | TCGA-AB-2924 | 3 | TCGA-AB-2981 | 3 |
| TCGA-AB-2823 | 3 | TCGA-AB-2874 | 3 | TCGA-AB-2925 | 2 | TCGA-AB-2982 | 1 |
| TCGA-AB-2824 | 2 | TCGA-AB-2875 | 3 | TCGA-AB-2927 | 3 | TCGA-AB-2983 | 1 |
| TCGA-AB-2825 | 3 | TCGA-AB-2876 | 1 | TCGA-AB-2928 | 3 | TCGA-AB-2984 | 1 |
| TCGA-AB-2826 | 1 | TCGA-AB-2877 | 1 | TCGA-AB-2929 | 1 | TCGA-AB-2985 | 2 |
| TCGA-AB-2828 | 1 | TCGA-AB-2878 | 3 | TCGA-AB-2930 | 3 | TCGA-AB-2986 | 2 |
| TCGA-AB-2830 | 3 | TCGA-AB-2879 | 3 | TCGA-AB-2931 | 2 | TCGA-AB-2987 | 3 |
| TCGA-AB-2832 | 2 | TCGA-AB-2880 | 3 | TCGA-AB-2932 | 2 | TCGA-AB-2988 | 3 |
| TCGA-AB-2833 | 2 | TCGA-AB-2881 | 3 | TCGA-AB-2933 | 1 | TCGA-AB-2990 | 3 |
| TCGA-AB-2834 | 1 | TCGA-AB-2882 | 3 | TCGA-AB-2934 | 2 | TCGA-AB-2991 | 1 |
| TCGA-AB-2835 | 2 | TCGA-AB-2883 | 1 | TCGA-AB-2935 | 3 | TCGA-AB-2992 | 3 |
| TCGA-AB-2836 | 3 | TCGA-AB-2884 | 3 | TCGA-AB-2936 | 3 | TCGA-AB-2994 | 1 |
| TCGA-AB-2837 | 3 | TCGA-AB-2885 | 3 | TCGA-AB-2937 | 3 | TCGA-AB-2995 | 3 |
| TCGA-AB-2838 | 3 | TCGA-AB-2886 | 3 | TCGA-AB-2938 | 3 | TCGA-AB-2996 | 3 |
| TCGA-AB-2839 | 1 | TCGA-AB-2887 | 3 | TCGA-AB-2939 | 3 | TCGA-AB-2998 | 3 |
| TCGA-AB-2840 | 1 | TCGA-AB-2888 | 3 | TCGA-AB-2940 | 3 | TCGA-AB-2999 | 1 |
| TCGA-AB-2841 | 2 | TCGA-AB-2889 | 3 | TCGA-AB-2941 | 3 | TCGA-AB-3000 | 3 |
| TCGA-AB-2842 | 3 | TCGA-AB-2890 | 1 | TCGA-AB-2942 | 1 | TCGA-AB-3001 | 1 |
| TCGA-AB-2843 | 2 | TCGA-AB-2891 | 3 | TCGA-AB-2943 | 3 | TCGA-AB-3002 | 3 |
| TCGA-AB-2844 | 3 | TCGA-AB-2892 | 1 | TCGA-AB-2944 | 3 | TCGA-AB-3005 | 2 |
| TCGA-AB-2845 | 3 | TCGA-AB-2893 | 2 | TCGA-AB-2946 | 3 | TCGA-AB-3006 | 3 |
| TCGA-AB-2846 | 3 | TCGA-AB-2894 | 1 | TCGA-AB-2948 | 3 | TCGA-AB-3007 | 1 |
| TCGA-AB-2847 | 3 | TCGA-AB-2895 | 3 | TCGA-AB-2949 | 3 | TCGA-AB-3008 | 3 |
| TCGA-AB-2848 | 1 | TCGA-AB-2896 | 2 | TCGA-AB-2950 | 3 | TCGA-AB-3009 | 3 |
| TCGA-AB-2849 | 1 | TCGA-AB-2897 | 1 | TCGA-AB-2952 | 2 | TCGA-AB-3011 | 1 |
| TCGA-AB-2851 | 3 | TCGA-AB-2898 | 3 | TCGA-AB-2954 | 3 | TCGA-AB-3012 | 1 |
| TCGA-AB-2853 | 3 | TCGA-AB-2899 | 3 | TCGA-AB-2955 | 1 |  |  |
| TCGA-AB-2854 | 3 | TCGA-AB-2900 | 3 | TCGA-AB-2956 | 2 |  |  |
| **Supplementary Information 2.** The distribution of common mutated genes was inconsistent in different clusters | | | | | | | |
| mutations | | Cluster 1 | | Cluster 2 | | Cluster 3 | |
| DNMT3A | | 10.0% | | 13.8% | | 11.2% | |
| NPM1 | | 5.0% | | 13.4% | | 10.1% | |
| TP53 | | 0.0% | | 13.8% | | 11.2% | |
| KIT | | 3.3% | | 6.9% | | 12.3% | |
| RUNX1 | | 13.3% | | 0.0% | | 9.0% | |
| FLT3 | | 5.0% | | 6.9% | | 4.5% | |
| IDH2 | | 6.7% | | 0.0% | | 5.6% | |
| WT1 | | 3.3% | | 3.4% | | 5.6% | |
| TTN | | 11.6% | | 3.4% | | 2.2% | |
| IDH1 | | 3.3% | | 0.0% | | 3.4% | |
